# Investigating perspectives and emotions on climate change among children and adolescents in San Cristóbal, Galápagos

**DOI:** 10.3389/fpsyg.2026.1935891

**Published:** 2026-10-06

**Authors:** Victoria Moffitt, Kelly P. Kennedy, Ayla Malefakis, Jennifer Pinto-Martin

**Affiliations:** 1 Perelman School of Medicine, University of Pennsylvania, Philadelphia, PA, United States; 2 Penn Global, University of Pennsylvania, Philadelphia, PA, United States; 3 Global Education and Research Alliance, Department of Philosophy, School of Arts and Sciences, University of Pennsylvania, Philadelphia, PA, United States; 4 School of Nursing, University of Pennsylvania, Philadelphia, PA, United States; 5 School of Arts and Sciences, University of Pennsylvania, Philadelphia, PA, United States

**Keywords:** adolescent mental health, climate change, climate change adaptation, climate change anxiety, climate education, eco-emotions, Galápagos

## Abstract

An estimated one billion children live at extremely high risk of adverse effects from climate change. Even when children are not exposed to direct physical impacts, the existential threat of climate change can have a psychological impact by creating a stressor that exacerbates underlying mental health disorders or contributes to the development of new disorders. Children and adolescents worldwide are experiencing high rates of fear and worry related to climate change, particularly in low- and middle-income countries. Despite this disparate impact, most studies on this topic have been conducted in the Global North. The Galápagos Islands are part of a medium-income nation and will face stark impacts of climate change in the coming decades. Currently, it is unknown how the threat of climate change is affecting the mental health of the children living in the islands. To address this gap, we conducted semi-structured, qualitative interviews with children and adolescents on the island of San Cristóbal to determine their thoughts, perspectives, and understanding of climate change (*N* = 30). These interviews also required participants to draw what they expect the island to look like in 100 years. We identified major themes from the interview transcripts and drawings, including the children’s personal identification with nature, feelings of anger and frustration, and feelings of sadness and loss regarding climate change. The findings from this study will inform interventions to promote emotional resilience among the children in San Cristóbal, as well as educational initiatives to address knowledge gaps.

## Background

### Climate change and its impact on adolescents

Children are particularly vulnerable to the physical and psychological impacts of climate change (Burke et al., 2018; Vergunst and Berry, 2022). Currently, 88% of the existing burden of physical disease attributable to climate change occurs in children younger than age five (Sheffield and Landrigan, 2011), and UNICEF reports that one billion children are living at “extremely high risk” of the impacts of climate change (UNICEF, 2021). Natural disasters intuitively threaten children’s mental health through risk of displacement and subsequent post-traumatic stress disorder (PTSD), depression, and anxiety (Burke et al., 2018). But even when children are not exposed to natural disasters, the existential threat of climate change can impact their mental health by creating a stressor that exacerbates underlying mental health disorders or contributes to the development of new disorders (Vergunst and Berry, 2022).

In the past decade, research into the notion of “climate anxiety” has emerged. This concept is defined as “anxiety associated with perceptions about climate change, even among people who have not personally experienced any direct impacts” (Clayton, 2020). It is important to note that climate anxiety is not a mental illness in and of itself; it may be considered a normal response to current events, and has even been associated with pro-environmental behaviors (Ogunbode et al., 2022). Nevertheless, climate anxiety has also been associated with decreased mental wellbeing scores and symptoms of mental illnesses, among both adults and children (Kuang and Root, 2019; Clayton and Karazsia, 2020; Ogunbode et al., 2022; Sciberras and Fernando, 2022; Reyes et al., 2023). Children and adolescents worldwide are increasingly worried about climate change, although this concern is highest in children in low- and middle-income countries (LMICs) (Boyes et al., 2014; Lee et al., 2020; Hickman et al., 2021). In an international survey of people aged 16–25 years, 84% of respondents were at least moderately worried about climate change, and 59% were very or extremely worried. Notably, 45% reported that their daily functioning was impacted by these feelings (Hickman et al., 2021). This is differentially distributed across the globe, with about 30% of children in the US, UK, and Australia reporting this daily impact, compared to about 70% of children in LMICs, such as India, Nigeria, and the Philippines (Hickman et al., 2021).

Despite this disparate impact, most studies on this topic have been conducted in the Global North; there is a current dearth of investigation into children’s perspectives and emotions toward climate change in LMICs, where 85% of children currently live (Burke et al., 2018; Lee et al., 2020; Crandon et al., 2022; Martin et al., 2022; Vergunst and Berry, 2022). These are the nations that will be most impacted by climate change, which is why numerous researchers recommend future studies in LMICs (Burke et al., 2018; Lee et al., 2020; Crandon et al., 2022; Martin et al., 2022; Vergunst and Berry, 2022), including research to better understand children’s awareness of climate change and how it is affecting their emotional wellbeing (Martin et al., 2022).

Investigations on the physical and mental health impacts of climate change in Latin America have been particularly limited; fewer than 4% of publications that pertain to climate and health focus on this region (Hartinger et al., 2024). A 2025 scoping review (Guarderas-Muñoz et al., 2025) of experimental and observational studies related to climate anxiety identified six pertinent publications focusing on populations in Latin America, four of which included adolescents living in Argentina, Guatemala, Chile, and Brazil (Chou et al., 2023; Rodríguez Quiroga et al., 2024; Sandoval-Díaz et al., 2024; Pinchoff et al., 2025). The remainder of the studies focused only on adult populations. In Ecuador specifically, there has been scarce investigation into this topic, especially considering the country’s emphasis on the value of the natural world. In fact, Ecuador’s 2008 Constitution designates rights and protections to nature itself and identifies response to climate change as a governmental priority (Ecuador, 2008). One study in southern Ecuador investigated adults’ emotional responses to climate change, and found that respondents in surveys and interviews most identified with negative emotional reactions, including concern, guilt, powerlessness, anger, and confusion (Iniguez-Gallardo et al., 2021). This study did not capture the perspective of children or adolescents, leaving the question of whether young Ecuadorians share these emotional responses to climate change, which is particularly important to investigate given the topic’s notable national significance.

### Climate change effects in the Ecuadorian province of Galápagos

Galápagos is one of 24 provinces in the middle-income country of Ecuador. Census data collected in 2022 (Instituto Nacional de Estadística y Censos, 2023) found a total of 28,583 residents living in the archipelago on four inhabited islands, divided into three municipalities. The capital of the Galápagos Province and the focus of our investigation is the town of Puerto Baquerizo Moreno in San Cristóbal Island, where 7290 individuals reside (Instituto Nacional de Estadística y Censos, 2023).

As outlined in the 2008 Ecuadorian Constitution, the Galápagos Province constitutes a *special system* and is thus overseen by the Government Council of the Special Governance System for Galápagos. The Council is chaired by the Minister of Galápagos—a representative appointed by the President of the Republic of Ecuador—and is comprised of the three mayors and other specified leaders of the region (Ecuador, 2008). Together, this Council retains regional jurisdiction over the archipelago, working in tandem with the Ecuadorian president to ensure the management of the islands proceeds in accordance with the nation’s constitution. Of note, the 1998 *Law on the Special Regime for the Conservation and Sustainable Development of the Galápagos Province* was enacted to control the management and development of the urbanized areas, prohibit further development of the islands, provide environmental protections for the archipelago, limit tourism and fishing, and to establish the Galápagos Marine Reserve. These protections were later bolstered by 2008 amendments to the Ecuadorian Constitution outlining the Rights of Nature, including four articles that provide legal pathways to both restore natural ecosystems and prosecute any individuals who conduct activities threatening renewable natural resources in the nation (Ecuador, 2008).

Galápagueños are a population particularly vulnerable to climate change. Socially, the community is homogenous with some gradation, but the smaller population in tandem with geographical distance from mainland Ecuadorians underscore social vulnerabilities related to physical isolation. Bureaucratic regulations impose further divisions; for instance, Ecuadorian citizens who are interested in relocating to Galápagos are frequently limited to a temporary residency, only offered to those who secure employment locally. Discrepancies exist in the physical and mental healthcare available to Galápagos residents compared to those in the continental provinces, where resources and infrastructure are more limited. While Galápagos is of higher socioeconomic status (SES) than mainland Ecuador due to its stable influx of tourism, it is considered a low-SES community compared to the United States (Instituto Nacional de Estadística y Censos, 2023).

Communities living in the Galápagos Islands will encounter dangerous impacts of climate change in the current century (Paltán et al., 2021). Mean annual precipitation will increase 30–45% by 2050 (Paltán et al., 2021) and El Niño climate events will occur with greater frequency and intensity, gravely threatening marine biodiversity (Dueñas et al., 2021). Extreme sea level events (which cause flooding due to storm surges, elevated mean sea level, and tides) that historically occurred once every 100 years (Oppenheimer et al., 2019) are expected to occur yearly by 2050, threatening coastal communities (Dueñas et al., 2021).

Children on the island are not currently experiencing natural disasters, but climate change poses threats to their natural environment, to culturally important species, and to their way of life in the coming decades. Their level of awareness and psychological impact from these threats is currently unknown.

### Exploring climate anxiety in the Galápagos Islands

The present investigation was guided by a conceptual framework in public health known as community based participatory research (CBPR). This framework is built on the idea that health is not simply an individual biological outcome but rather is shaped by social, economic, environmental and cultural condition. CBPR rests on the assumption that people possess knowledge about their own lives and lived experience that may not be captured in conventional interviews or questionnaires. The *draw-and-explain* technique changes the traditional relationship between researcher and subject and engages the community as a co-producer of the knowledge. The technique can reveal how someone conceptualizes a health or environmental issue and what it feels like and means to them. This gives the community members a way to communicate knowledge that may otherwise be difficult to articulate.

This project aims to understand children’s perspectives, emotions, and understanding of climate change in San Cristóbal, Galápagos. The findings from this work will inform the creation of a survey to capture children’s emotional responses to climate change that can be used internationally, fulfilling a need identified in the literature (Martin et al., 2022). It will also inform the community’s climate change adaptation planning and the development of educational initiatives around climate change in accordance with the Ecuadorian Ministry of Education’s new Contextualized Curriculum for Galápagos (Ministerio de Educación, 2021). These efforts may help mitigate the impact of climate change on the psychological wellbeing of children in LMICs.

## Methods

### Study design and interview approach

This project emerged from 5 years of collaborative community research performed by the Global Education and Research Alliance (GERA), which was co-founded by a Galapagueño naturalist guide and lawyer in San Cristóbal and two United States professors. Additionally, this study was designed to address concerns raised by community members and both local and national Ecuadorian authorities in meetings with GERA. GERA agreed to work alongside community members and local government officials to support Galapagueños in their climate change adaptation planning and school curriculum design.

This qualitative interview study combined draw-and-explain activities (Alerby, 2000; Shepardson et al., 2011; Strife, 2012) with semi-structured interviews to elicit children’s perspectives and emotions regarding their natural environment and the potential future impact of climate change. A similar draw-and-explain technique was used successfully in past studies to solicit children’s perspectives on the environment (Alerby, 2000; Shepardson et al., 2011; Strife, 2012). Our design asked children to draw and discuss what they think San Cristóbal will look like in 100 years. The 100-year time frame was selected to allow children to reflect on the legacy of their natural world beyond their own lifetimes, considering a timeframe that implicates future generations. Studies have shown that young people have particularly high concerns regarding climate impacts beyond mid-century, and this has been known to trigger anxiety in young people as they consider important life choices, such as the decision to have children of their own (Miller, 2018; Morning Consult, 2020; Schneider-Mayerson and Leong, 2020). A prior study among Ecuadorian adults found that their concerns evoked by climate change stemmed from apprehension around the future and a sense of legacy, making this an important angle to investigate among youth as well (Iniguez-Gallardo et al., 2021). Additionally, the 100-year projection also allows for greater alignment with climate science prediction models by the IPCC, which use the year 2100 as an anchor for many of its projections of sea level rise that will directly impact islands like San Cristobal. Although the children are not expected to be familiar with this data specifically, using this timeframe allows us to contextualize their interpretations of the future within the realities of scientific predictions, which currently suggest more frequent extreme sea level events will double or triple annual flood damages in coastal communities by 2100, compounded with exacerbating habitat contraction and loss of biological diversity (Oppenheimer et al., 2019). While the conclusion of the 21st century is technically closer to ∼80 years in the future, the 100-year threshold was considered a simpler benchmark for children to conceptualize.

Discussing animals is also an effective facilitator for children to share their thoughts on the environment (Alerby, 2000; Paul Hoggett, 2019; Wiggin et al., 2021). In the interviews, they were asked about local animals and the future of their environments, about their daily lives and interactions with nature, and to envision timepoints in their own future. Afterwards, they were asked directly about climate change, where they learned about it, and their messages for adults on this topic (for complete interview guide, see Supplementary material). Interview duration was 15–30 min, not including drawing time. Interviews were conducted either in the participant’s home, at a GERA office, or in another mutually-agreed-upon location. All interviews were conducted in Spanish, the language most frequently spoken by Galapagueños. Interviews were facilitated by a public health graduate researcher who had cultural familiarity with Galápagos (VM).

Three pilot interviews were conducted with children ages 8, 12, and 17 in San Cristóbal in March 2022, all of whom were members of the Scout troop. Minor modifications were made to the interview guide prior to conducting formal interviews in June and July 2022.

### Sample recruitment

The target population consisted of children aged 7–17 years who lived on the island of San Cristóbal, Galápagos; meeting these age and residency requirements were the two inclusion criteria. This age range was selected to detect differences in perceptions that may exist across children in different age groups. The minimum age of 7 years was chosen in consultation with a child psychologist as the likely lower age limit for effective engagement in the interview. Demographics collected include age, gender, number of years lived on the island, parental professions, and affiliations to GERA extracurricular programs. Parental professions are used as a descriptor of SES, although imperfect, and for our purposes were used to provide insight on the educational background at home.

Participants were recruited using convenience sampling, targeting children with prior affiliations with GERA activities, and through snowball sampling from GERA’s community partners. Children in the local all-gender scout troop, Scouts Oceánicos Independientes de Galápagos (Scouts), were invited to participate in interviews, as well as children engaged in a marine science initiative with GERA. Additionally, one of GERA’s local partners, a pastor of an Evangelical church, facilitated access to the children of this faith community for study participation. GERA also participated in an environmental fair outside of the town’s municipal building in which the project was briefly described and interested parents could sign up to hear more about the project and receive an official invitation to participate. The fair was mainly frequented by school children, but a few parents did attend, and one signed up to hear more. The children’s parents/guardians and the children themselves were invited to participate either in person or through WhatsApp messages. They were told that the research project was designed to understand how children in San Cristóbal think and feel about their environment and climate change. If they were interested, an interview was scheduled, and the consent/assent process took place immediately before each interview. The official recruitment message was sent to 58 potential participants via their parents: 40 were Scouts, 14 were divers, three were recruited through the Evangelical church, and one was recruited through the environmental fair. Thirty-two children and their parents agreed to undergo the informed consent process. After reviewing the informed consent and assent documents, two participants did not undergo the interview. In one case, this was a potential participant recruited through the Evangelical church and the parents refused to proceed due to fear that the child’s responses would somehow be traced back to the parent. In the other case, the potential participant was recruited through the environmental fair and the child refused due to discomfort with being recorded. Of the original 58 individuals recruited for this study, our final sample included 30 children (52%).

### Institutional review board

This study received approval by two Institutional Review Boards (IRB): one at the University of Pennsylvania (Protocol # 851131) and a local IRB in-country, through the Comité de Ética de Investigación en Seres Humanos de Universidad San Francisco de Quito (CEISH-USFQ; code IE02-EX035-2022-CEISH-USFQ).

### Data analysis

Interviews were recorded with participant assent and guardian consent and were transcribed prior to analysis. Three interviews (10%) were analyzed initially using inductive open coding by two coders (VM, AM) to create a preliminary codebook. This codebook was further discussed and modified through meetings with a qualitative methods expert (JC) and the principal investigator (JPM). Clear definitions, criteria for uses, and exceptions were detailed in the completed codebook. Both coders then used these criteria along with NVivo software to code 20% of the interview transcripts, after which minor adjustments and additions were made to the codebook. An additional 20% of the transcripts were coded followed by a calibration meeting in which coding discrepancies were discussed and consensus was reached. The remaining interviews (50%) were coded by the primary coder (VM).

The associated drawings for each interview were similarly analyzed, with features of each image coded within NVivo. Intercoder reliability for the interview transcripts was evaluated and produced a Kappa coefficient of 0.98 and average percent agreement of 99.84%. Although a Kappa coefficient could not be calculated within the NVivo software for the drawings, the coders discussed and reached agreement for each image. With consensus reached on the correct application of the codebook, the remainder of the drawings and images were coded by VM, who also wrote memos reflecting on the perspectives shared verbally by each participant during their interviews (see Supplementary material).

All coded data was then organized into themes, which distilled the content from all interviews and drawings into the most salient topics. Various NVivo queries were used to further analyze the data. Coding queries and crosstab queries were used to assess for differences in the frequency with which sentiments were expressed across different demographic groups and to assess text with overlapping themes.

## Results

### Participants

We conducted interviews with 30 participants. Demographics of the participants are presented in Table 1. The median age of participants was 14 years (range = 8–16). The gender distribution consisted of 13 boys and 17 girls. Twenty-one of the participants were members of the Scouts, two were recruited from an Evangelical church, and seven were affiliated with GERA’s marine science and scuba diving initiative. The small sample size of the church group relative to the Scouts group does not allow for meaningful comparative analysis.

**TABLE 1:** Participant demographics.

Type	Details	N	% of total (*N* = 30)
Gender	Boys: 13 (43%)	13	43%
	Girls: 17 (57%)	17	57%
Age	8–10 years	4	13%
	11–12 years	7	23%
	13–14 years	6	20%
	15–16 years	13	43%
Time living in San Cristóbal	Whole life or moved age < 3	26	87%
	Moved at age ≥ 8	4	13%
	Of those who moved at age ≥ 8, moved within past three years	2	7%
Recruitment group	Scouts	21	70%
	Divers	7	23%
	Church	2	7%
Parental professions*	STEM	8	n/a*
	Tourism agency	7	
	National park/naturalist guide	5	
	Retail	5	
	Construction/Carpentry	4	
	Government/Legal	4	
	Teacher	3	
	Church	2	
	Captain/Fisherman	2	
	Farmer	2	
	Seamstress	1	

*Total does not add up to *N* = 30 and percentages are not provided because many participants had two parents with two different occupations.

The children participating in GERA’s scuba diving activity were intentionally recruited from schools that served a community with low socioeconomic status (SES), so this extracurricular affiliation served as a proxy for low SES. The most common parental profession was in STEM; nine participants (30%) had a parent with a job in math or sciences, including engineers, accountants, and meteorologists. Five of the children interviewed (17%) had a parent who was a naturalist guide or worked in the National Park, five participants had at least one parent who worked in tourism agencies, and five participants had parents who worked in retail. See Figure 1 for a complete depiction of the parents’ occupations. Twenty-six participants (87%) had lived in San Cristóbal either for their whole lives or had moved there before they were 3 years old. Four participants moved to the island at age eight or older; two of these four had lived on the island for at least 6 years, and two had moved within the past 3 years.

**FIGURE 1:**
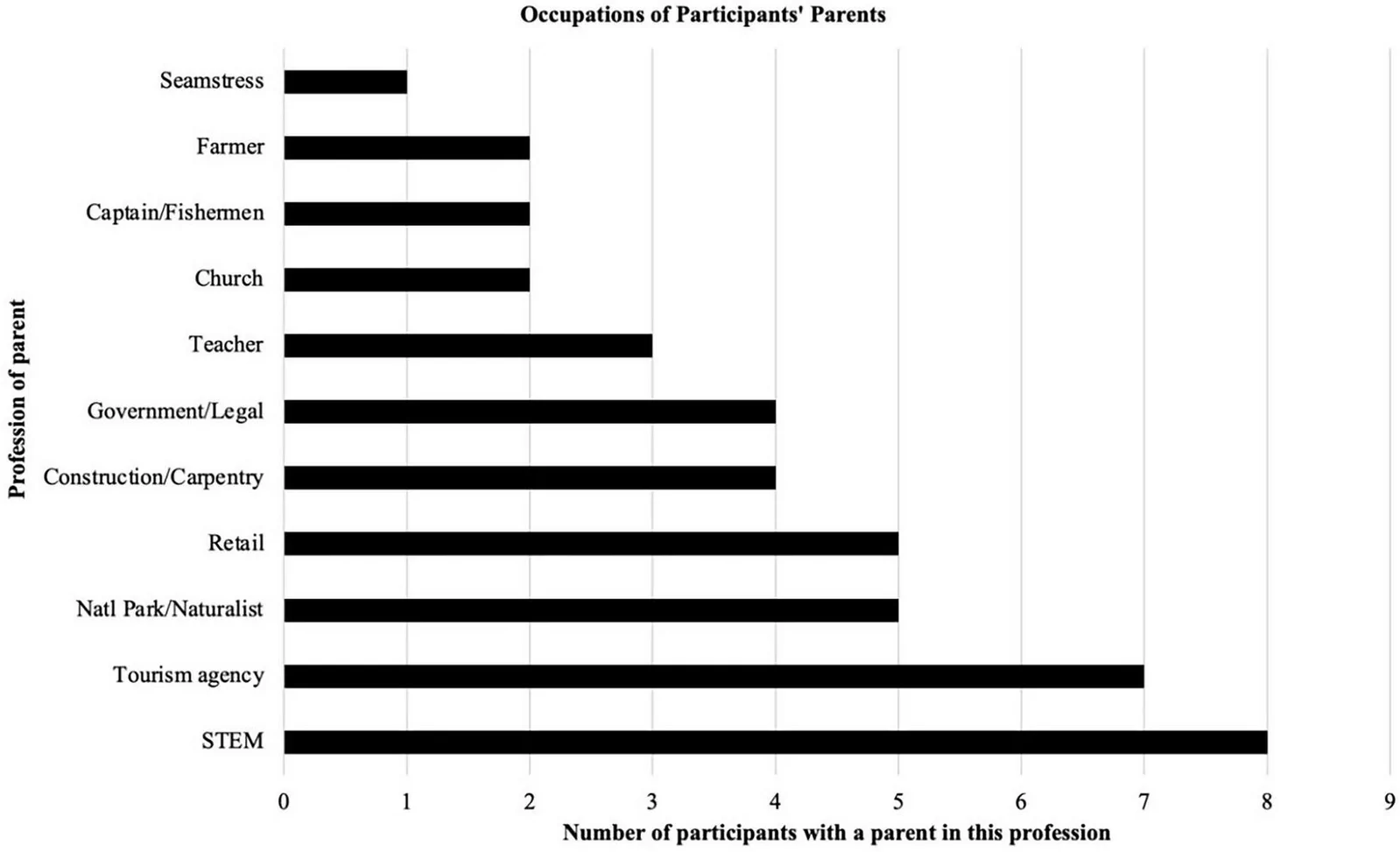
Occupations of participants’ parents. The total number of participants indicated by the columns is greater than 30 because many participants had two parents with two different occupations.

All quotes provided in the sections below have been translated from Spanish. They are each cited with the participant’s assigned number (#1–30) followed by the age and gender of the participant. Table 2 organizes the quotes by theme and subtheme and includes many additional examples that are not included in the body of the text.

**TABLE 2:** Participant quotes organized by theme.

Theme	Participant quotes, organized by subtheme
**Theme:** animal concerns	**Subtheme: personal connection to animals** “¡*Ah! San Cristóbal is beautiful. I love when the sea lions are posing, the little sea lions that are in the sand. I also like seeing the sea turtles swim, the manta rays. The animals, I love all that, the species that are here. Also, the highlands; there are beautiful birds. The frigate birds.”* (Participant #18, 8-year-old girl) *“I hear many sea lions [when walking around San Cristóbal]. I hear iguanas*…*they make this sound on the sand when they are stepping. And from their footprints and tail*…*One time I asked my dad what those lines are [in the sand]. And he said that they were from the iguana tails.”* (Participant #10, 8-year-old girl) *“We tend to walk along the [waterfront]. We tend to go to the dock, and stay there watching the ocean and the sea lions and all of that.”* (Participant #25, 13-year-old girl) *“When I went diving, in Tijeretas, I remember that once I found, because once I saw a starfish. I was at least around 12 years old and for me it was amazing to see a starfish*…*and I saw turtles like that too and more sea lions. Sea lions scared me at first and lately this year I’ve gotten over it. Now when I see them [when diving] I just stay still and calm myself down, but I was scared. Now I just see them and then I calm down.”* (Participant #14, 14-year-old girl)
	**Overlap with Nature, Community, and Identity Theme** *“Several times that I’ve been, that important things have happened, I’ve seen manta rays. When I was in Isabela one time with my dad, we saw manta rays. When it was an important dive, I saw a manta ray. When I went to León Dormido* [a famous rock formation in the ocean]*, a manta ray. So it’s like something that, that is always there, when it is something important.”* (Participant #16, 15-year-old girl) *“Well, I have an appreciation for the natural world. Because I don’t know, to see all those animals coexist, to be free. To see, sometimes their facial expressions are a bit funny, let’s say when they eat or do things.”* (Participant #1, 15-year-old boy) *“That is something that characterizes the island a lot. They are animals that can’t be found in other places. I mean, maybe yes, but they are unique.”* (Participant #12. 16-year-old girl) *“We have tons of vegetation as well as fauna. And well, the fauna here is very endemic as well as native, as is the flora.”* (Participant #11, 14-year-old girl) *“[Sea lions] are very representative of*… *of*… *how people are too, a little bit here in the islands, so*…*they are very playful. They love to go out, they swim a lot and Galápagos is sometimes represented with the sea lions.”* (Participant #29, 15-year-old boy) *“[Sea lions] represent us.”* (Participant #15, 16-year-old boy)
	**Overlap with emotional responses** “*Another concern is that if the island were to become abandoned, without animals or anything, it would no longer be a unique place, it would no longer be a natural heritage of humanity.”* (Participant #11, 14-year-old girl) *“Galápagos would be nothing without [animals]. The island is maintained by them. And also, if it weren’t for —many years ago, Charles Darwin. He came to these islands and he discovered finches and evolution, he discovered that here. Also. if there were no animals on this island, it wouldn’t be an island, it would be something abandoned, with nothing of value. Nothing. There wouldn’t even be people because in the end, if [animals] leave, all the people leave.”* (Participant #14, 14-year-old girl) *“The animals are going to become extinct. Yes, because the turtle is under conservation status. The sea lions now have conservation status, but there are not many anymore. And the iguanas are at risk of disappearing.here, when change occurs, global warming, the El Niño phenomenon that we call it, uh, the animals of the Galápagos suffer a lot to get food. For example, iguanas have to travel a little deeper than they should have to get algae. Baby sea lions have, sometimes some, I saw a report that some sea lions are dying from waiting for their mother, because they have to dive deeper to find food.”* (Participant #13, 14-year-old girl) *“The species. Because*…*as I’ve said, they’re unique. And if we think about when – in the case where [the animals] end up disappearing, the island would be totally*…*.it would alone. There would be so much silence, which on this island is very rare. You can always hear sea lions or birds, all types of things. Without that, it would be too much silence.”* (Participant #12, 16-year-old girl) *“[Climate change is] very sad. Yes, very sad. Because, as it is in the north pole, they also support us. Imagine how they are melting and they have life there, for example, the polar bears. Imagine what a terrible way to die, drowning when everything melts.”* (Participant #13, 14-year-old girl) *“Many species are going extinct lately. Like the polar bear because of climate change. As you can see over there, the poles are melting a lot. And all those things. And I have seen videos and they even kill each other for food, and they are very skinny, compared to what they were before. And I am very concerned about polar bears, because their home is melting, and it becomes less, less.”* (Participant #14, 14-year-old girl)
**Theme:** nature, community, and identity	*“Where I live, it’s a place that is very*…*very environmental because we are surrounded by flora and fauna. Yeah! And different from the rest of the world. Because the habitat of Galápagos is very impressive, compared to other environments.”* (Participant #6, 12-year-old boy) *“I think we were talking about something very important. Which is that, which is more important than technology and what is more important is nature, because it gives us this*…*everything we need, food, oxygen, it gives us the oxygen that we need so much. Even shelter.”* (Participant #5, 10-year-old male) *“Honestly, when I was little, I didn’t even know that Galápagos existed. And because of my family*… *I didn’t want, I didn’t want to come and live here. It was really hard for me to get used to. Yes, it’s been some years, and now, because of school and my friends*…*they helped me a lot to get used to it and the nature [helped] too, because it’s very beautiful and I’m sincerely lucky to live here.”* (Participant #19, 16-year-old girl) *“We should think that nature is very beautiful. It’s very special. Each thing has, I mean, it is very special. Very different.”* (Participant #9, 11-year-old girl) Subtheme: Peace and Belonging in Nature *“I look at the horizon of the ocean, at the furthest point, as if the ocean was going to end. I concentrate there, alone, having peace, a connection with the ocean. But if I go back to see another part [of the island], I see only cars, only city, only town.”* (Participant #11, 14-year-old girl) *“When I go out for a walk with my friends*… *we go at night, we go to the beach and it feels really nice, listening to the waves and the ocean. The sunset as it goes down, disappearing between the sea and the sky. To feel the pure air, the fresh air. It also feels very nice when it rains. Being there, in the rain, standing still.”* (Participant #19, 16-year-old girl) *“I think [nature] is like my place of comfort. It’s a place where I feel safe, where I can walk calmly. And also, I love it here. Here it is like a paradise to be, a paradise for me, because I am lucky to live here. Because look, if one day I’m angry, stressed, I go to the beach, I relax, and I come back and have it close. There are people who don’t even know what a beach is.”* (Participant #14, 14-year-old girl) *“It is a relaxing experience [to be in nature]. Because you disconnect from any concern and relax. And, you feel the environment together with you as if it were another part of your body.”* (Participant #6, 12-year-old boy) *“I sometimes have fun [outside]. Because I start to get distracted. Now, now if I get stressed, which happens to me. If I’m stressed, uh, I start doing something* *[outside] and I no longer think about that thing that stresses me out.”* (Participant #23, 16-year-old boy) *“It’s super relaxing*… *you feel calm, you separate yourself a bit from the daily routine.”* (Participant #24, 15-year-old boy) *“The foam of the ocean crashing against the stones, and it sounds something like [imitates sound]. The smell of the sea. It’s what I love most about living here.”* (Participant #15, 16-year-old boy)
	Subtheme: experiencing nature with family “*Well, on my dad’s birthday, during the pandemic, we went to the beach, Cabo de Hornos. The ocean was so beautiful and we were relaxing and all that, watching the clouds.”* (Participant #15, 16-year-old boy) *“It relaxes me. I mean, if I have tons of worries and homework, among other responsibilities. When I’m alone or with family in the open air, it’s as if nothing bothers me, I feel free*…*more relaxed. All of your problems go away.”* (Participant #19, 16-year-old girl)
	Overlap with emotional reponses “*I think [climate change] is something that shouldn’t be happening, that is something that, that is happening to us because people—not everyone, because yes, there are people who fight—are being bad with the climate, because we should protect it, instead of harm it. Because we are one with it, because nature is what makes us. We would be nothing, truly [without it]. We would be like in misery, in ruin.*” (Participant #14, 14-year-old girl) *“I have mixed feelings because everything here seems very beautiful to me. The fact that, in reality, none of our ecosystems are ours. We are like a plague in this world. And plagues always survive, they always find a way to survive. So, continuing the same way*…*other living beings dying along the way. And the truth is I have mixed feelings and everything because, yeah, everything is going to change.”* (Participant #4, 13-year-old girl)
**Theme:** Pollution	**Participants explaining litter in their drawings** “*The people who only litter killed [the animals*…*They think it’s food and they are going to choke on the garbage. And they choke and are going to die.”* (Participant #10, 8-year-old girl) *“I see two options in the future depending on how we act in the present. That is to say, if people decide to take care of the islands and protect them, we will have a situation like this* [the positive drawing]*, and it will continue to be preserved. In the other drawing I am showing what happens if the people mistreat it.”* (Participant #14, 14-year-old girl)
	**Subtheme:** **understanding of climate change** *“As we know well. Today, climate change is a reality, there is no denying it, and it is caused by us. And as long as more people go taking garbage and throwing it in our oceans, there is going to be more climate change*…*A long time ago, [climate change] didn’t exist and it is because of the litter, because of the aerosols, because of the factory fuels, because of the factory smoke, because of the cars. Also, because of, also cow excrement is a pollutant.”* (Participant #14, 14-year-old girl) *“[Climate change is caused by] burning fuels or fossils, uh, dumping garbage on beaches. A lot of fishing, well yes, not respecting the closed seasons, because here the closed seasons are very respected*… *well, what is happening right now, as I already said, a lot, a lot of cars with smoke, the factories, there is a lot of plastic waste, which is killing [animals*]*.”* (Participant #15, 16-year-old boy) *I’d like to say [to adults]*…*that please don’t litter on planet earth, because if not, it’s going to get worse. And well, if they keep doing it, well, well, it’s going to cause earthquakes, tsunamis, earthquakes, storms, tornados. And imagine that, if one day you cut down the trees every day, every day and the trees run out. We wouldn’t have fresh air and we would be breathing gases and things like that.”* (Participant #10, 8-year-old girl) *“The litter doesn’t help this island much because the big ocean surges will come and the docks could collapse and there won’t be anything from San Cristóbal anymore.”* (Participant #2, 12-year-old girl)
	**Subtheme:** **taking action** *“In the case of adults and those of us who are young people, we can encourage the little ones and those of the whole community to*… *um, learn how to use*… *recycling, caring for nature, not throwing garbage wherever because that is why there are trash bins*… *um*… *Yes, some people, if they ride a bicycle so as not to be using, so that gas does not come out of cars and those polluting things. I say that we must raise awareness, have more, if we don’t know, investigate more on the subject about the consequences, that we are going to have, in the*… *it could be now or in the new generations that*… *we should reconsider. Because if not, these islands are, they are wonderful. You can say a paradise. And if we don’t take care of it now, what will the new generations receive, if we don’t do things right, the new generations won’t do it either. And little by little the islands will be lost.”* (Participant #19, 16-year-old girl) *“In my message [to adults about climate change] it would be that, to your children, the ones who are going to keep living here, what do you teach them*…*You can teach them from a young age to save trash. To be able to teach them about it so they don’t cause the same thing and help the population*… *Uh, yes, I would say that the mayor here in San Cristóbal, or we, who are a group of children in the Scouts, can help, go to different houses and help them and teach them about climate change*…*We can do recycling campaigns and we can prevent all this happening.”* (Participant #2, 12-year-old girl) *“[Adults] should teach all their generations not to pollute. They should learn*… *They shouldn’t be so proud, if a person tells you to learn not to pollute this planet, the planet. Because if we only took care of this island*…*It’s not going to work, because the earth is going to expel us, try to expel everyone. We have to take care of the whole world.”* (Participant #18, 8-year-old girl)
	Overlap with nature, community, and identity theme “*Everyone has the*…*or, almost everyone teaches us*… *according to me, the custom that we have to take care of our animal species in the Galápagos. No littering and all that stuff. Because we are always taught in school. that is, we are always taught that we have to be more conservationist*… *[In the future, I think the island will be] very modernized. But I hope that everyone applies that, I don’t know, that how shall we say, culture of. of conservationism that they have always taught us and do not occupy natural spaces.”* (Participant #1, 15-year-old boy) *“Pollution. There is a lot of talk about pollution, more than anything here on the islands we are always made aware that pollution is what causes all of this.”* (Participant #29, 15-year-old boy) *“I talk a lot about climate change here. If you walk along the waterfront or along a street, you meet people who talk about it. They give you a brochure, they give you a quiz where you can answer questions.”* (Participant #29, 15-year-old boy) *“Well, most of the beaches I’ve seen are usually very clean, but there are certain people who are quite irresponsible and leave their trash behind. I’d say that, for a few years, after many years, I believe that it can be changed. people can change and become aware of the harm that we are doing to our, to our Island.”* (Participant #19, 16-year-old girl)
**Theme:** Emotional responses	**Subtheme: sadness and loss** *Describing her drawing of an abandoned island: “It doesn’t have flora, not a bit of fauna either, [so] there would be no tourism there*… *It’s realistic because it’s what we are living. We are not living in a world of happiness.”* (Participant #11, 14-year-old girl) *“Some things that I think about, when I go see the sunsets and I see that one day this is going to end*…*These islands are like the treasure here. And one day there will be none of this. And then our memories will stay in the past.”* (Participant #2, 12-year-old girl) *“The trees will no longer have more*… *more leaves and everything will be littered. The clouds will no longer be white; they will be gray and there won’t be much sun anymore. Because everything will be polluted and everything is dark. And the sky is all contaminated and. And the ground will be like this, cracked and dry. And there will be bad, bad drought, and there will be no more water.”* (Participant #3, 11-year-old girl). *“I think of the generation that would come after me and everything they have to see and what they will not be able to see: color and birds and everything related to the island. It’s sad.”* (Participant #12, 16-year-old girl) *“[Climate change] is bad. It’s sad because climate change is changing so much. And I don’t want the beautiful animals of Galápagos to go extinct.”* (Participant #18, 8-year-old girl) *“I feel sorry, because everyone, not just us, is affected. but also the animals. It’s not their fault.”* (Participant #21, 11-year-old girl) *“I would say [to adults] that they should become aware of the steps they are taking and more, not only for themselves, but also for their descendants. Because, even if they die and don’t get to see a, a very ugly planet. Their children, their grandchildren. If they ever see it, it won’t be a good life for them.”* (Participant #26, 14-year-old boy)
	**Subtheme: fear and worry** *“We have to be worried [about climate change] because this is gradually being lost. And suddenly there will be nothing. And there won’t be any more food, and there will be many losses, and cutting down trees. And well, we have to be very worried about the climate.”* (Participant #27, 15-year-old boy) *“Sometimes [climate change] scares me*…*like something bad is going to happen, but I don’t know when it will be.”* (Participant #25, 13-year-old girl) *“[Climate change] makes me sad. And it also scares me because, I mean, it hurts people and animals more than anything. It will destroy everything, practically everything.”* (Participant #20, 15-year-old girl) *“Well, I hope that*…*it gets better, because I wouldn’t want to live in a world where it’s, uh, like in the movies, they use masks to receive oxygen. I wouldn’t like that world, none of that.”* (Participant #15, 16-year-old boy) *“There are many people, well, many young people, and in my case, I would not like, for example, to have children at the moment. One, because I am young, and two, also because I think that. if there are people who are not going to educate their children, this is going to happen and they are going to suffer all these things. So I think it’s going to be something very awful for my children that they suffer things from climate change that are going to come very soon.”* (Participant #16, 15-year-old girl) *“Climate change not only affects animals or fish, it also affects us. And yes, there has been talk about it, there has been talk about how it affects us in breathing and sooner or later it will not only affect what we see, but ourselves. Our breathing.”* (Participant #29, 15-year-old boy) *“100 years is a long time. It’s supposed to, well, according to me and according to the things I have heard, they think that in 100 years there will be no more human life. Because of everything that climate change has been going through. In 2030 I think they had said that we are already going to see various difficulties in the world. So, in 100 years I think that it will no longer exist, Galápagos would no longer exist.”* (Participant #16, 15-year-old girl)
	**Subtheme: frustration and anger** “*A message for adults about climate change*…*Instead of just giving words, why don’t you give actions? In other words, they say, ‘We are going to do it,’ but they don’t, they just talk, they just talk all the time, they just want to talk and not listen to others. And as an adolescent, young person, child, you cannot say anything because they are the authority at that moment. So they do not want or do not want to hear an opinion from someone less than them and they want to be superior*.” (Participant #11, 14-year-old girl) *“Well, my feelings are, like, a little angry and a little sad. Because despite. despite what they know is going to happen. That there will be a day when they cannot get into the water. One day there is going to be a big, big elevation of the sea. And it can cause harm to many, to different people here in the town that are near the water. And also, the animals that I like, I mean, they would die. And the feeling of anger is because they know what is going to happen and they do not do something to help.”* (Participant #2, 12-year-old girl) “*My feelings [about climate change]? As you can imagine, what I feel is anger, truly, because it is irresponsibility. And because one person cannot change everything; one person could change everything, at the same time that another irresponsible person says that they’re going change, but they don’t do it. It makes me angry.”* (Participant #11, 14-year-old girl) *“I think [climate change] is a very difficult situation*…*.I think it is almost impossible to teach people to be conscientious about what we are doing. So it’s somewhat complicated for me, I think that’s what I was saying to my dad, if we as Ecuador affect 1% of the environment and 99% are not going to be able to do anything, we as Ecuador are not going to be able to do anything. Because there are millions of people, it is very difficult for only Ecuador to be able to do something.”* (Participant #16, 15-year-old girl) *“We have to stop being like this. We have to open our eyes, we have to think about the other species, we have to take care of the environment, not continue building large buildings*…*Climate change is caused by people who don’t take care for the environment*…*We pollute, and the Earth must try to expel us, by harming itself.”* (Participant #18, 8-year-old girl) *“We know that this is serious, because [the adults] always tell us that we have to take care of [the environment]. But, the next day they go and litter. Like this, in a place, seriously, because it won’t just be us [who is affected], but them.”* (Participant #21, 11-year-old girl)
	Subtheme: optimism “*I imagine a San Cristóbal with less garbage in the ocean and more populated and that is equally calm and safe, with more marine species.”* (Participant #30, 15-year-old girl) *“I say that people will see a change, at least in Galápagos. They say at school a lot that people are watching us, people have their sights on us, so we always try. Not long ago I think they tried to expand the marine reserve and I think people will also want to continue to see that. A clean environment in Galápagos.”* (Participant #29, 15-year-old boy) *“In 100 years. I feel that, if projects are started here, and well, and people also become aware that they shouldn’t [litter] because they harm nature.”* (Participant #30, 15-year-old girl) *“There will always be someone who wants to make a difference.”* (Participant #12, 16-year-old girl) *“I imagine that [in 30 years], while carrying out construction, more restaurants, more jobs, premises and I think that, with the care that we can give to nature, we can have more, more flora and fauna on these islands.”* (Participant #19, 16-year-old girl) *“Even though as the years go by on the island, there will still be people. But for example, the National Park protects spaces for plants or animals. And if in the future, technology advances, it can make, for example, flying cars and also marine garbage collectors, to take it to a specific place to process it.”* (Participant #6, 12-year-old boy) *“The dangers that the environment has faced have already been reducing thanks to the authorities.”* (Participant #6, 12-year-old boy)
	Subtheme: frequency of thoughts about climate change “*[I think about climate change] constantly. Because it is something that is happening a lot and you see so much of how it’s being demonstrated in earthquakes, that some countries have been flooded, that the sea level is rising. That animals are dying, so it’s something that you see very constantly.”* (Participant #1, 15-year-old boy) *“[I think about climate change] almost all the time*…*because they say that you can live on other planets that are being discovered, as well as live on another planet. But. I think that if they discover that there is a way to live on other planets, it will not be possible because it is not the same as Earth.”* (Participant #13, 14-year-old girl) *“[I think about climate change] almost always. Because on the Internet we can see, for example, anything in videos that YouTube recommends. It shows you about what is currently happening.”* (Participant #6, 12-year-old boy) *“[I think about climate change] like every day. Because here you always know, there is a lot of sun.”* (Participant #18, 8-year-old girl) “*I don’t have much time to think about so many things that go through my mind.*” (Participant #11, 14-year-old girl) *“Not every day, but when I see someone do something bad. Sometimes I even do some bad thing, but I remember that I shouldn’t do it, because of the teachings they have given me and the care I have to take in the Scouts; we value nature very much, and the life of each being.”* (Participant #19, 16-year-old girl) *“[I don’t think about climate change] as much as before. But now, like once a month. Two times. Recently, it was at León Dormido* [a famous rock formation in the ocean].” (Participant #26, 14-year-old boy) *“[I think about climate change] when it’s a subject I study at school.”* (Participant #24, 15-year-old boy)

### Themes

Across different age groups, backgrounds, and genders, certain perspectives and topics were consistently raised by the participants in their interviews. These topics constitute the major themes of the data: Animal Concerns; Nature, Community, and Identity; and Pollution. While discussing these topics, the participants shared varying Emotional Responses, which serves as an additional theme. The drawings reflected these central themes as well, and features were categorized thematically.

Drawings were also categorized as positive, negative, or neutral depictions of the future. This classification was determined based on both the drawings themselves and on the participants’ explanations of their drawings. Negative drawings displayed pollution, litter, dead or distressed animals, or loss of human life. Positive drawings depicted improvements to the environment with indications of humans and nature living in harmony. Neutral drawings either depicted the island as it is now without any death or contamination drawn, or with minor changes to the island such as increased construction and urban expansion without harmful environmental impacts. Of the 30 total drawings, 19 (63%) were classified as negative depictions, five (17%) were classified as neutral depictions, five (17%) were classified as positive depictions, and one contained two alternate futures: one positive and one negative. Figures 2–4 contain examples of each drawing type. Figure 2 is an example of a negative depiction drawn by a 16-year-old girl: it displays a tree with a sad face growing out of a pile garbage, with factories emitting fumes in the distance. A bird is drawn dead in the air, and sea lions in the ocean cry out for help. Notably, however, the participant said she drew the person sitting on the mound to indicate that “*there will always be someone who wants to make a difference*.” Figure 3 is an example of a neutral depiction drawn by a 14-year-old boy: it features Playa Mann, the most frequented beach on the island; he drew more buildings than are currently present, but the beach is otherwise unchanged. A sea lion sleeps safely, and human beachgoers are present. Figure 4 is an example of a positive depiction drawn by a 12-year-old boy: it displays an expanded national park labeled as *Area Protegida* (Protected Area), with humans happily working to defend nature and helping it to thrive, as depicted by healthy flowers and cacti. A garbage-collecting drone (*recolectador de basura marina*) flies overhead to remove litter from the ocean.

**FIGURE 2:**
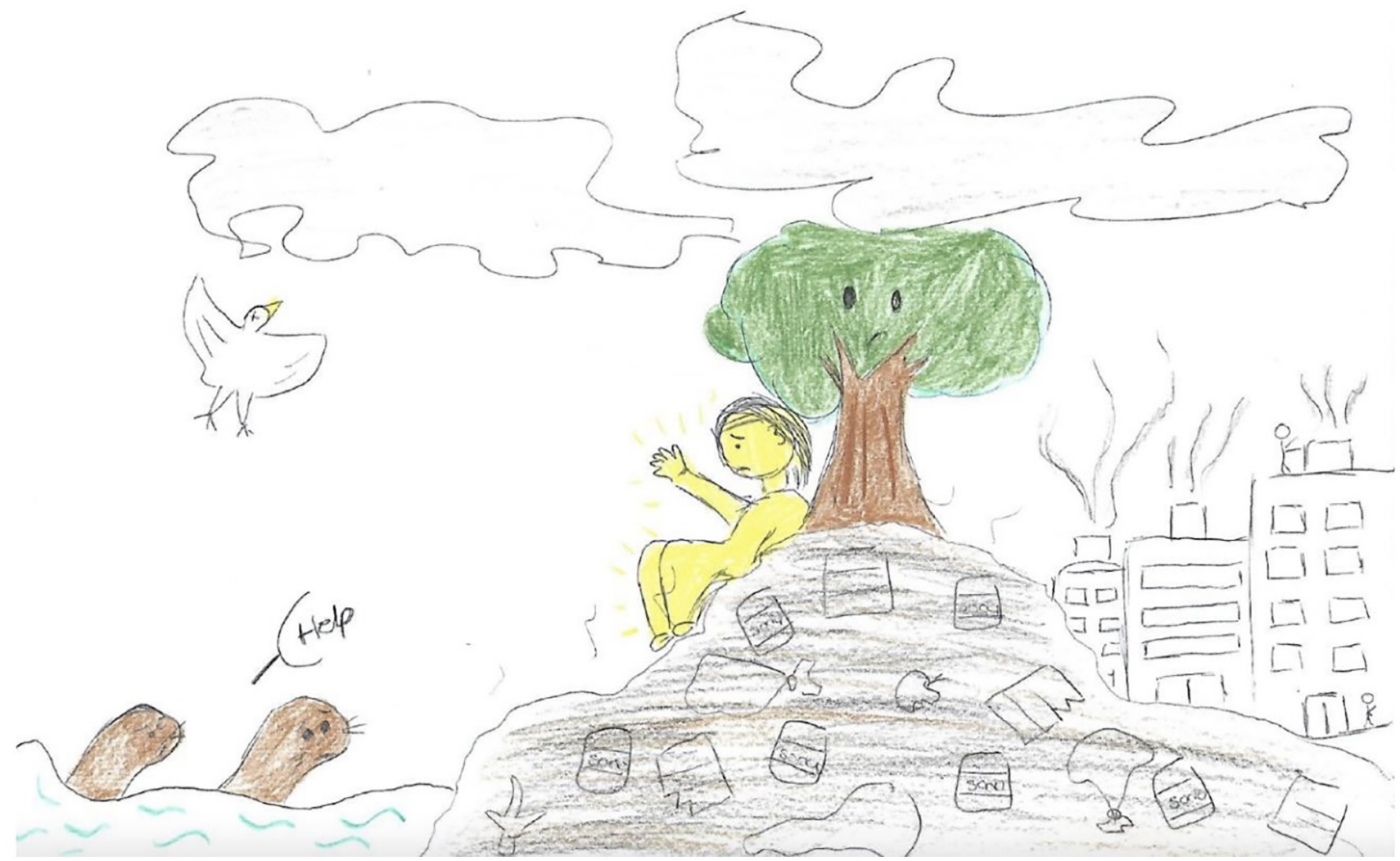
Drawing by participant #12, 16-year-old girl. Negative depiction of future.

**FIGURE 3:**
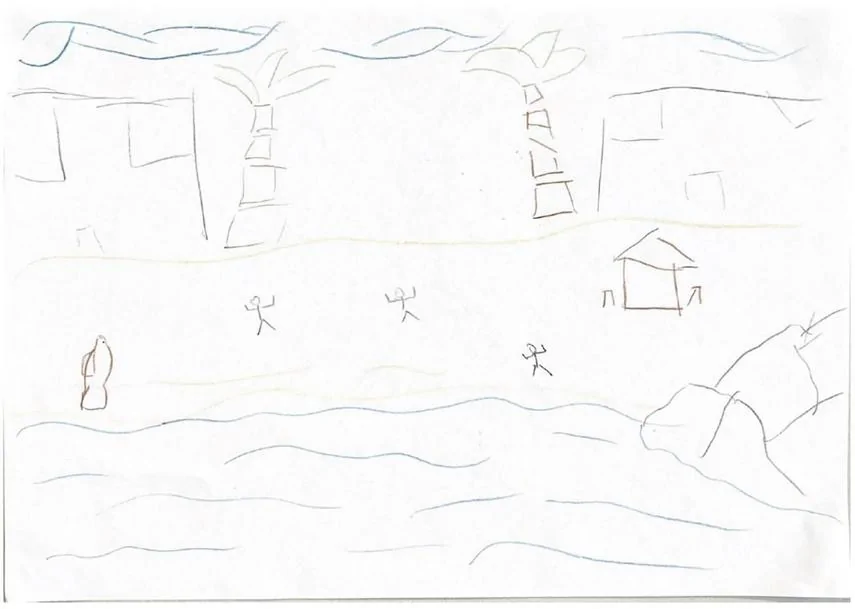
Drawing by participant #24, 14-year-old boy. Neutral depiction of future.

**FIGURE 4:**
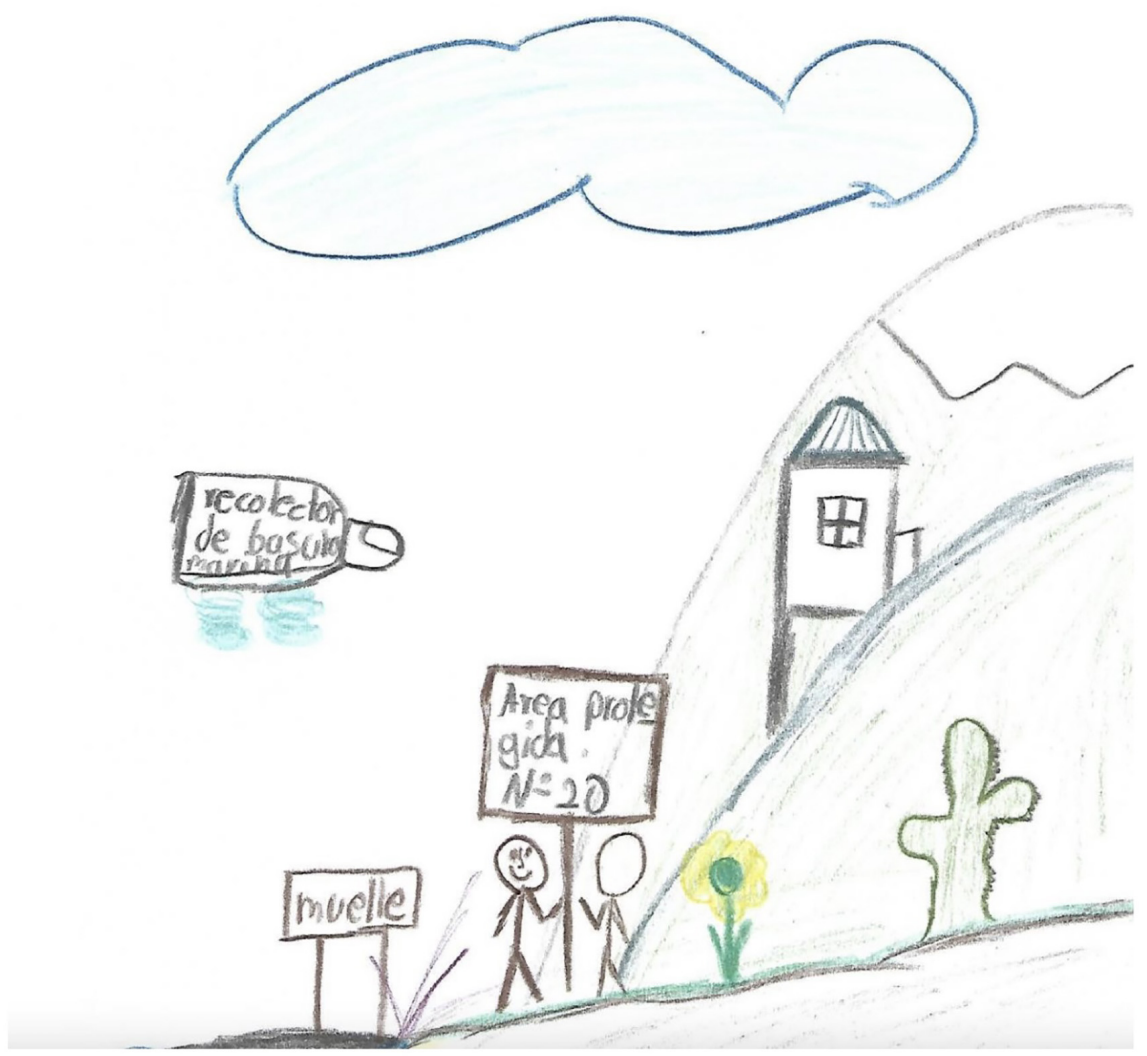
Drawing by participant #6, 12-year-old boy. Positive depiction of future.

There were differences in the distribution of drawing types across genders. Fourteen (83%) of the 17 girls drew negative depictions compared to five (38%) of the 13 boys. Two girls (12%) drew positive drawings, and 3 boys (23%) drew positive depictions. All five of the neutral drawings belonged to boys. The one drawing that featured two possibilities for the future (one positive and one negative) belonged to a girl. There was also variation among the different recruitment groups. Fourteen (67%) of the 21 Scouts drew negative depictions, compared to three (43%) of the seven divers. An additional three (43%) of the divers drew neutral depictions compared to only two (9%) of the Scouts. The two groups had comparable proportions of positive drawings, with one diver (14%) and four Scouts (21%) drawing optimistic depictions. One of the Scouts made the drawing with two halves, one positive and one negative. The remaining two participants who were recruited through an Evangelical church both drew negative depictions.

There were differences in drawing patterns between those with various parental professions, as well. Amongst those who had a parent who works in STEM or as a naturalist guide, nine (75%) drew negative depictions of the future, two (17%) drew positive depictions, and one (8%) drew a neutral depiction. Among those whose parents had other jobs that would most likely be susceptible to climate change impacts (Farmers, Captain/Fishermen, Tourism Agency), three (27%) drew negative pictures, two (18%) drew positive pictures, and four (36%) drew neutral pictures. One of those three negative drawings belonged to a child who also had a parent who worked as a naturalist guide, creating one point of overlap between the two groups of professions described above.

Analyzing the drawings across different age groups, 50% of the participants that were age 14 to 16 (8/16) drew negative depictions of the future compared to 71% of those age 12 to 13 (5/7), and 100% of those age 8 to 11 (7/7). The youngest participant with a positive drawing was age 12.

#### Animal concerns

Animal Concerns were very prominent in the children’s perspectives, not only when they considered the future of the island and the impacts of climate change, but also when they reflected on their daily lives. Every participant (100%) identified an animal on the island that they felt was important to them and was able to explain why. Many participants expressed great love for the animals that they see day to day, and described how encountering them is part of their normal routines:

“¡*Ah! San Cristóbal is beautiful. I love when the sea lions are posing, the little sea lions that are in the sand. I also like seeing the sea turtles swim, the manta rays. The animals, I love all that, the species that are here. Also the highlands; there are beautiful birds. The frigate birds.” (Participant #18, 8-year-old girl)*

One of the subthemes of this category was Personal Connection to Animals, which tended to overlap with the Nature, Community, and Identity theme; 13 participants (43%) shared sentiments that combined these two themes. For example, many children identified strongly with local animals, or even perceived that certain animals accompany them during important life moments.

*“Several times that I’ve been, that important things have happened, I’ve seen manta rays. When I was in Isabela one time with my dad, we saw manta rays. When it was an important dive, I saw a manta ray. When I went to León Dormido* [a famous rock formation in the ocean]*, a manta ray. So it’s like something that, that is always there, when it is something important.”* (Participant #16, 15-year-old girl)

As part of the same overlapping themes, many participants asserted that the animals are crucial to the essence and identity of their island. Galápagos is home to many endemic species (species that can only be found in these islands), which is a fact that the children (27%) frequently raised.

*“That is something that characterizes the island a lot. They are animals that can’t be found in other places. I mean, maybe yes, but they are unique.”* (Participant #12, 16-year-old girl)

Nearly a quarter of the children (23%) expressed the connection between animals and the island’s identity along with Emotional Responses toward climate change and the future.

*“Galápagos would be nothing without [animals]. The island is maintained by them. And also, if it weren’t for —many years ago, Charles Darwin. He came to these islands and he discovered finches and evolution, he discovered that here. Also. if there were no animals on this island, it wouldn’t be an island, it would be something abandoned, with nothing of value. Nothing. There wouldn’t even be people because in the end, if [animals] leave, all the people leave.”* (Participant #14, 14-year-old girl)

This island identity was particularly entwined with the sea lions. Interview responses featured *lobos marinos*, or sea lions, prominently. San Cristóbal is the island in Galápagos with the greatest concentration of sea lions, and they are almost always visible along the *malecón* (waterfront), sleeping and swimming at the docks, main beaches, and main walkways of town. Even when they are not visible, sea lions still command attention on the *malecón* due to their frequent, and therefore ubiquitous, vocalizations. When asked about the typical sights, sounds, and smells of San Cristóbal, most participants mentioned sea lions. In fact, 28 of the participants (93%) discussed them, while 10 of the drawings (33%) included at least one sea lion. When asked if there is an animal on the island that is important to them, one third of participants chose the sea lion. As one participant explained, this is “*because they represent us*” (Participant #15, 16-year-old boy). Another participant expressed the same sentiment:

*“They are very representative of*… *of*… *how people are too, a little bit here in the islands, so*…*they are very playful. They love to go out, they swim a lot and Galápagos is sometimes represented with the sea lions.”* (Participant #29, 15-year-old boy)

This affinity for sea lions often translated into a concern for their future. In the drawings of the future of San Cristóbal, seven participants (23%) depicted the sea lions dying, threatened, or in distress. Figure 5 displays a drawing from a 14-year-old girl that depicts a “Museum of Galápagos from 100 years ago,” which she imagined would be built to honor the wildlife that would be extinct in the future. Inside, she drew images of a sea lion crossed out to indicate that the species had gone extinct, alongside tortoises, iguanas, and endemic plants. Figure 6, from a 13-year-old girl, shows a sea lion choking and dying on a littered candy wrapper, surrounded by a trash-strewn beach. Other drawings, Figures 7, 8 depict sea lions with cramped habitats that have been encroached upon by urbanization.

**FIGURE 5:**
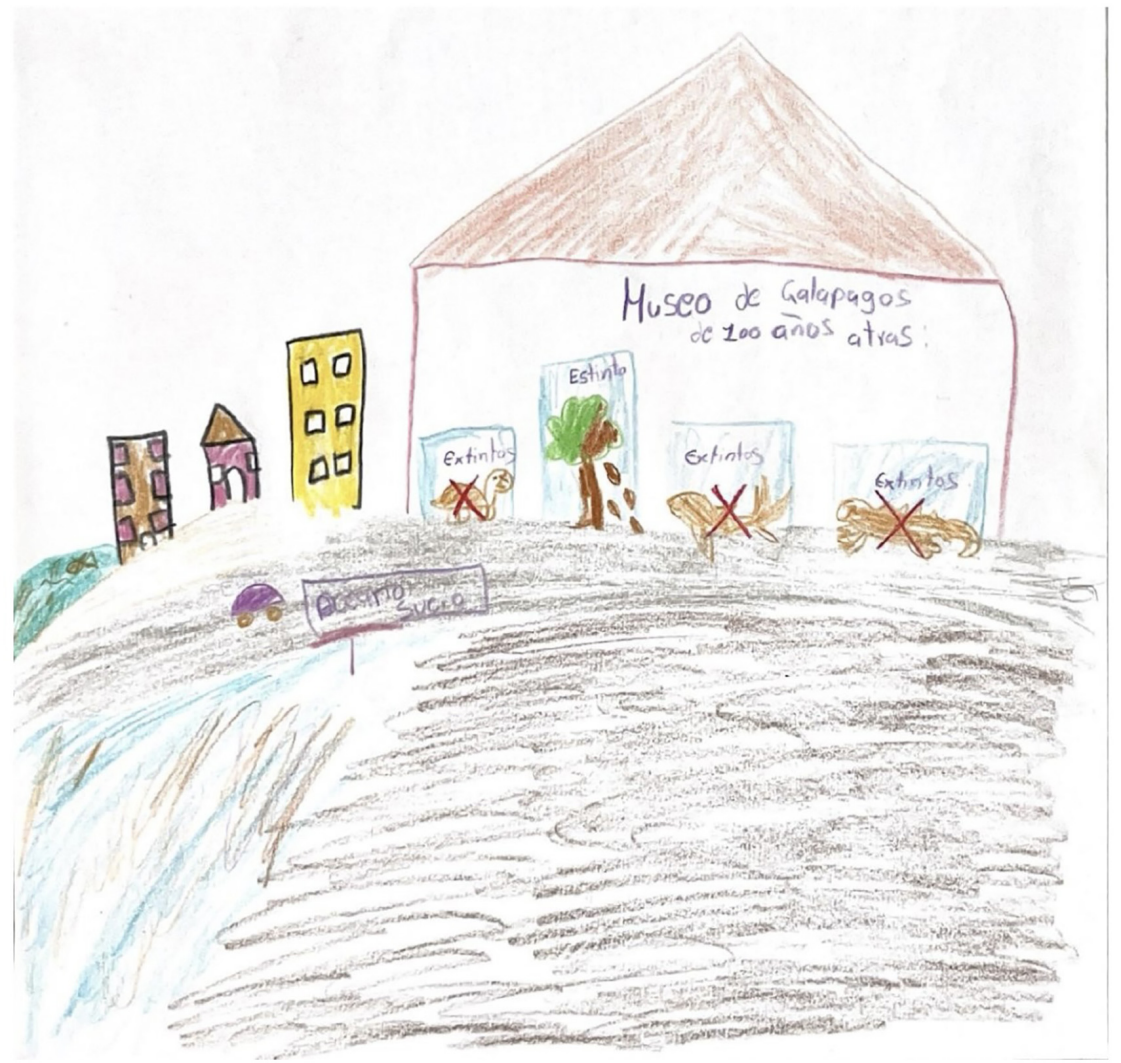
Drawing by participant #13, 14-year-old girl. Negative depiction of future.

**FIGURE 6:**
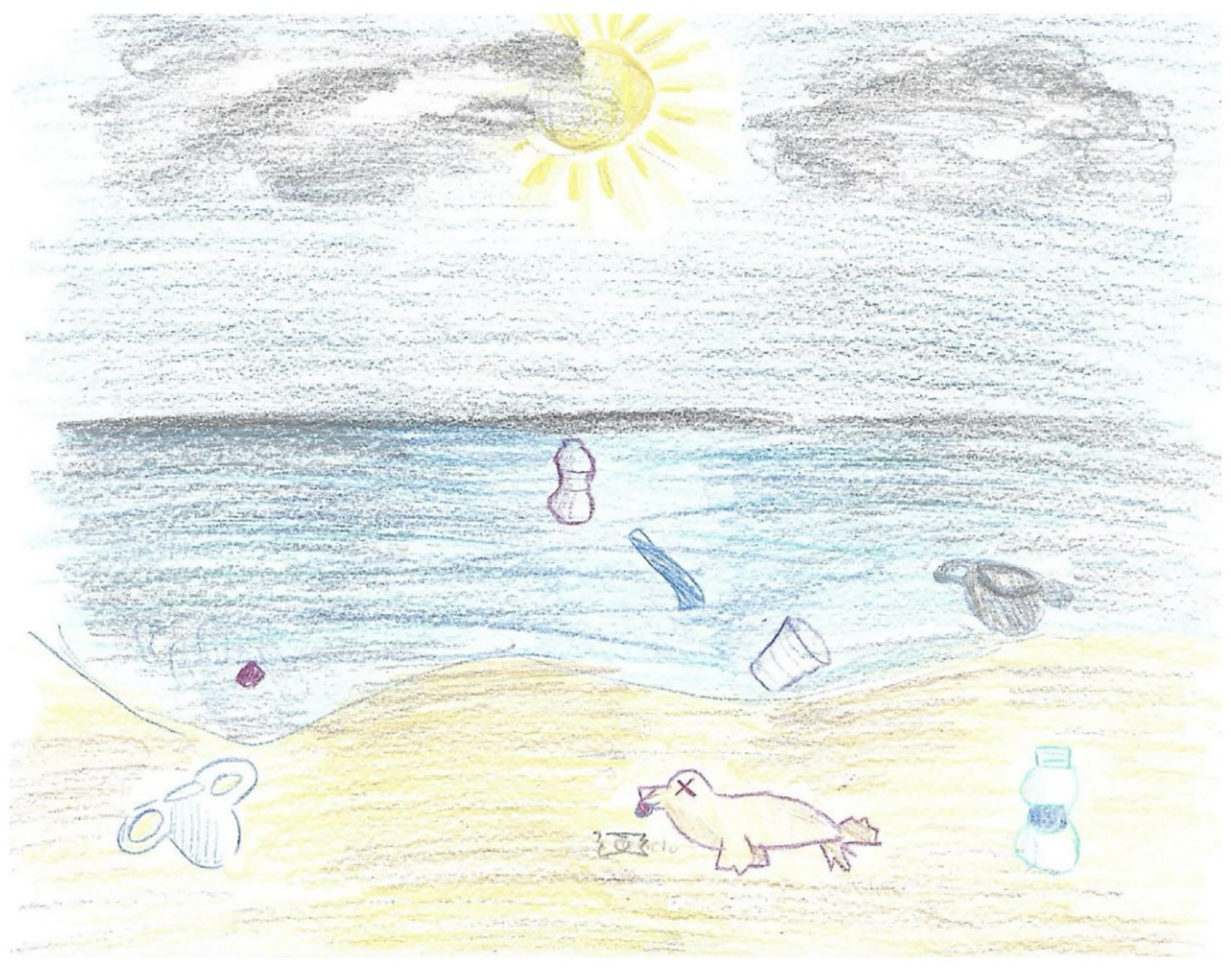
Drawing by participant #4, 13-year-old girl. Negative depiction of future

**FIGURE 7:**
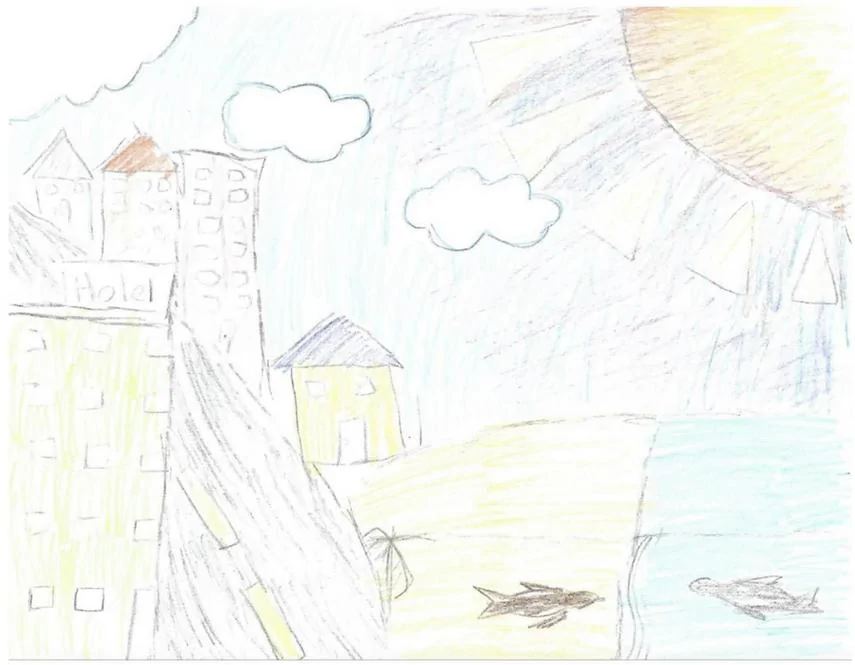
Drawing by participant #8, 12-year-old girl. Negative depiction of the future.

**FIGURE 8:**
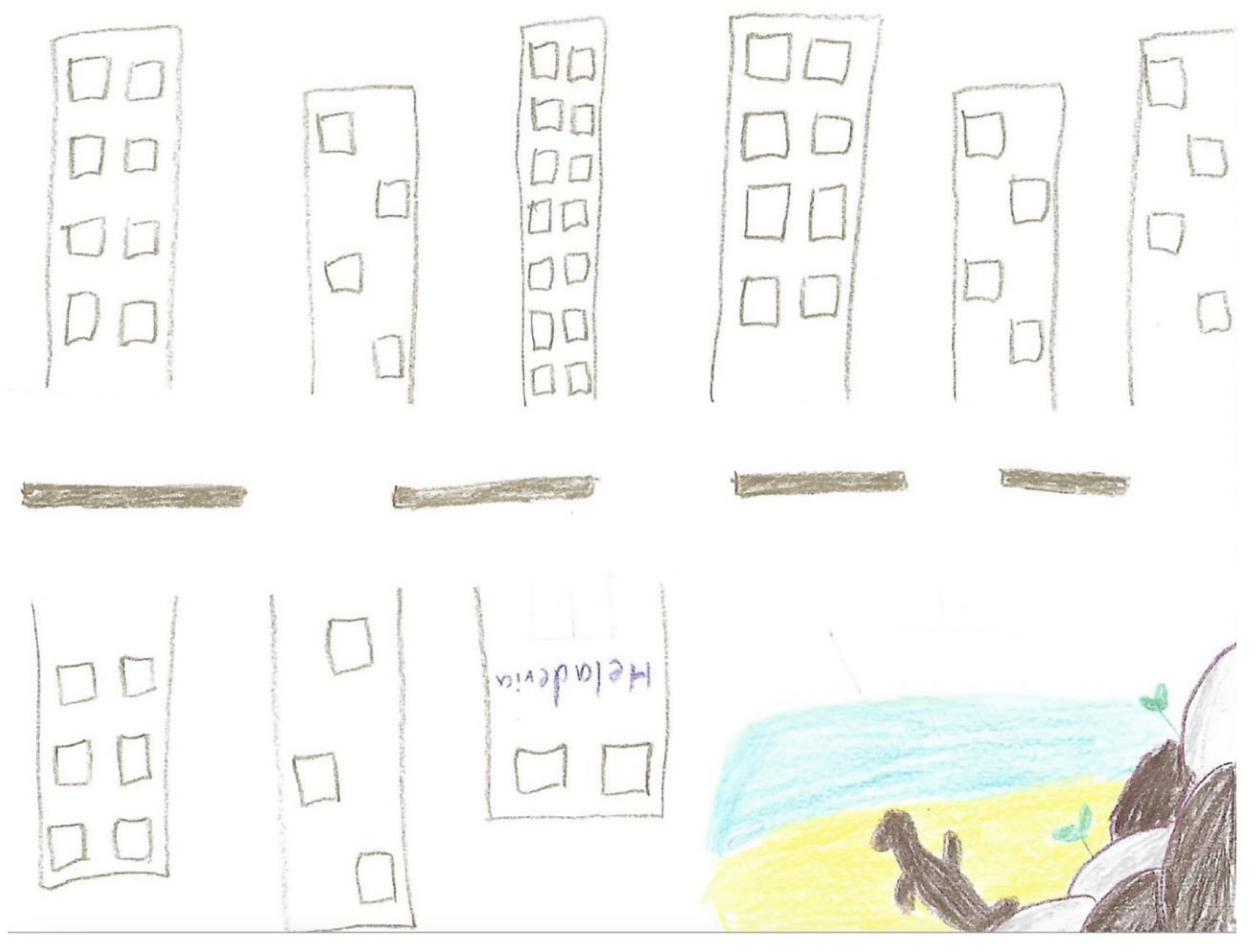
Drawing by participant #9,11-year-old girl. Negative depiction of the future.

Every participant except for one (97%) discussed a concern that many animals would die and animal species would be reduced in the future due to pollution and climate change. Most participants (77%) also expressed Emotional Responses to the future when discussing their concerns for animals.

*“The animals are going to become extinct. Yes, because the turtle is under conservation status*…*.The sea lions now have conservation status, but there are not many anymore. And the iguanas are at risk of disappearing. Here, when change occurs, global warming, the El Niño phenomenon that we call it, uh, the animals of the Galápagos suffer a lot to get food. For example, iguanas have to travel a little deeper than they should have to get algae. Baby sea lions have, sometimes some, I saw a report that some sea lions are dying from waiting for their mother, because they have to dive deeper to find food.”* (Participant #13, 14-year-old girl)

Several participants even expressed concern for species of animals that they had never seen before. Five participants (20%) discussed the threats to polar bears due to climate change. Whether they were discussing local animals or those that live in distant regions, most interviews (16/30, 53%) included discussions of animal loss framed with tones of sadness and loss:

*“The species. Because*…*as I’ve said, they’re unique. And if we think about when – in the case where [the animals] end up disappearing, the island would be totally*…*.it would be alone. There would be so much silence, which on this island is very rare. You can always hear sea lions or birds, all types of things. Without that, it would be too much silence.”* (Participant #12, 16-year-old girl)

*“[Climate change is] very sad. Yes, very sad. Because, as it is in the north pole, they also support us. Imagine how they are melting and they have life there, for example, the polar bears. Imagine what a terrible way to die, drowning when everything melts.”* (Participant #13, 14-year-old girl)

These concerns were shared by participants of all ages, but the two 8-year-old participants discussed Animal Concerns more than any other age group, particularly discussing human actions harming animals. They shared discussions about Personal Connection to Animals more than any other age group as well.

#### Nature, community, and identity

In addition to the identification with animals described above, 25 participants (83%) identified more broadly with nature. They often took pride in the island itself.

*“Where I live, it’s a place that is very*…*very environmental because we are surrounded by flora and fauna. Yeah! And different from the rest of the world. Because the habitat of Galápagos is very impressive, compared to other environments.”* (Participant #6, 12-year-old boy)

Additionally, participants expressed sentiments related to Peace and Belonging in Nature, a common subtheme. Twenty-seven participants (90%) expressed a sense of relaxation or calm in nature, explaining that being in nature reduces their stress and soothes them. In 20% of interviews, this overlapped with another subtheme, Experiencing Nature with Family.

*“It relaxes me. I mean, if I have tons of worries and homework, among other responsibilities. When I’m alone or with family in the open air, it’s as if nothing bothers me, I feel free*…*more relaxed. All of your problems go away.”* (Participant #19, 16-year-old girl)

*“I think [nature] is like my place of comfort. It’s a place where I feel safe, where I can walk calmly. And also, I love it here. Here it is like a paradise to be, a paradise for me, because I am lucky to live here. Because look, if one day I’m angry, stressed, I go to the beach, I relax, and I come back and have it close. There are people who don’t even know what a beach is.”* (Participant #14, 14-year-old girl)

Similar to participants’ concerns about losing animals to pollution, urbanization, and climate change, many participants expressed Emotional Responses towards the loss of nature in the future. Most participants (25/30, 83%) shared expressions of sadness and loss with regards to the future of the environment and climate change:

*“I think [climate change] is something that shouldn’t be happening, that is something that, that is happening to us because people —not everyone, because yes, there are people who fight—are being bad with the climate, because we should protect it, instead of harm it. Because we are one with it, because nature is what makes us. We would be nothing, truly. We would be like in misery, in ruin.*” (Participant #14, 14-year-old girl)

*“I have mixed feelings because everything here seems very beautiful to me. The fact that, in reality, none of our ecosystems are ours. We are like a plague in this world. And plagues always survive, they always find a way to survive. So, continuing the same way*…*other living beings dying along the way. And the truth is I have mixed feelings and everything because, yeah, everything is going to change.”* (Participant #4, 13-year-old girl)

This theme varied notably between participants with different backgrounds. The Scouts had many more statements coded to this theme than other groups. In the subthemes of Experiencing Nature with Family, the code showed up more than twice as often among Scouts than among Divers or other participants. There was a similar distribution of coding for the Peace and Belonging in Nature subtheme, with Scouts again having more than twice as many references to this topic compared to those recruited through the church, and about 1.5 times as many references compared to the Divers. The subtheme Experiencing Nature with Family was especially frequent among the two 8-year-old participants, who had about twice as many references to this topic when compared to the rest of the sample.

#### Pollution

Nearly all participants made references to garbage, pollution, and littering in their interviews (29/30). Litter was heavily featured in the drawings of the future as well, with 12 drawings (40%) featuring garbage. These drawings often depicted devastating consequences of littering. Figure 9 shows a drawing from an 8-year-old girl, which depicts littered masks on the ground and X’s over the trash cans to demonstrate that no one is using them. She said there were no animals in the drawing because “*the people who only litter killed them*…*[the animals] think it’s food and they are going to choke on the garbage. And they choke and are going to die”* (Participant #10, 8-year-old girl). The clouds in the drawing have sad faces, which she said was “*because they see everything is polluted.”* Above the tree in her drawing, she wrote, “*There is only 1 tree*,” to indicate that there is only one tree left in the world, and the rest had been cut down.

**FIGURE 9:**
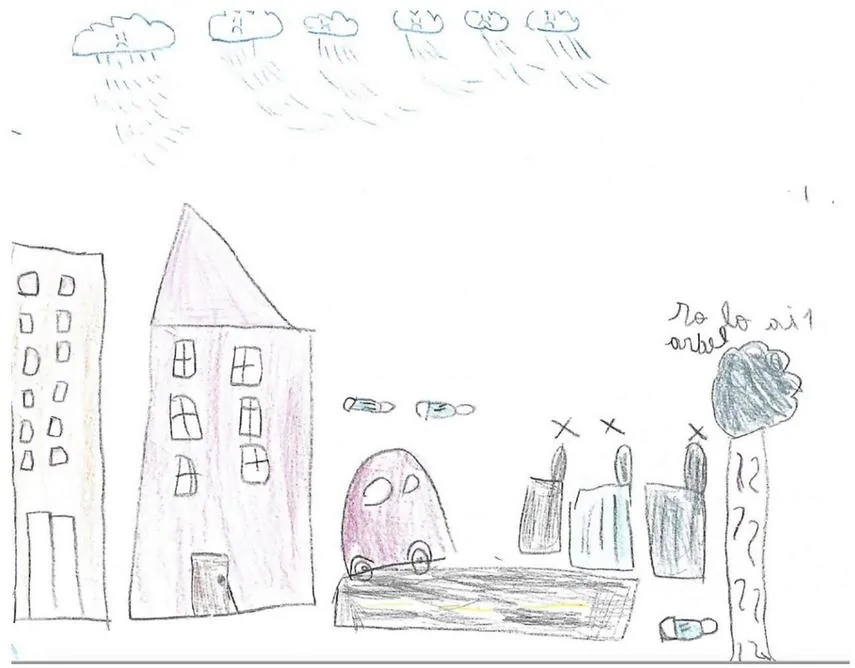
Drawing by participant #10, 8-year-old girl. Negative depiction of the future.

Figure 10 depicts a drawing by a 14-year-old girl that is split down the middle: one half, a heavily polluted beach with garbage and oil spills, with many animals choking on garbage; the other, clean with smiling animals. She explained,

**FIGURE 10:**
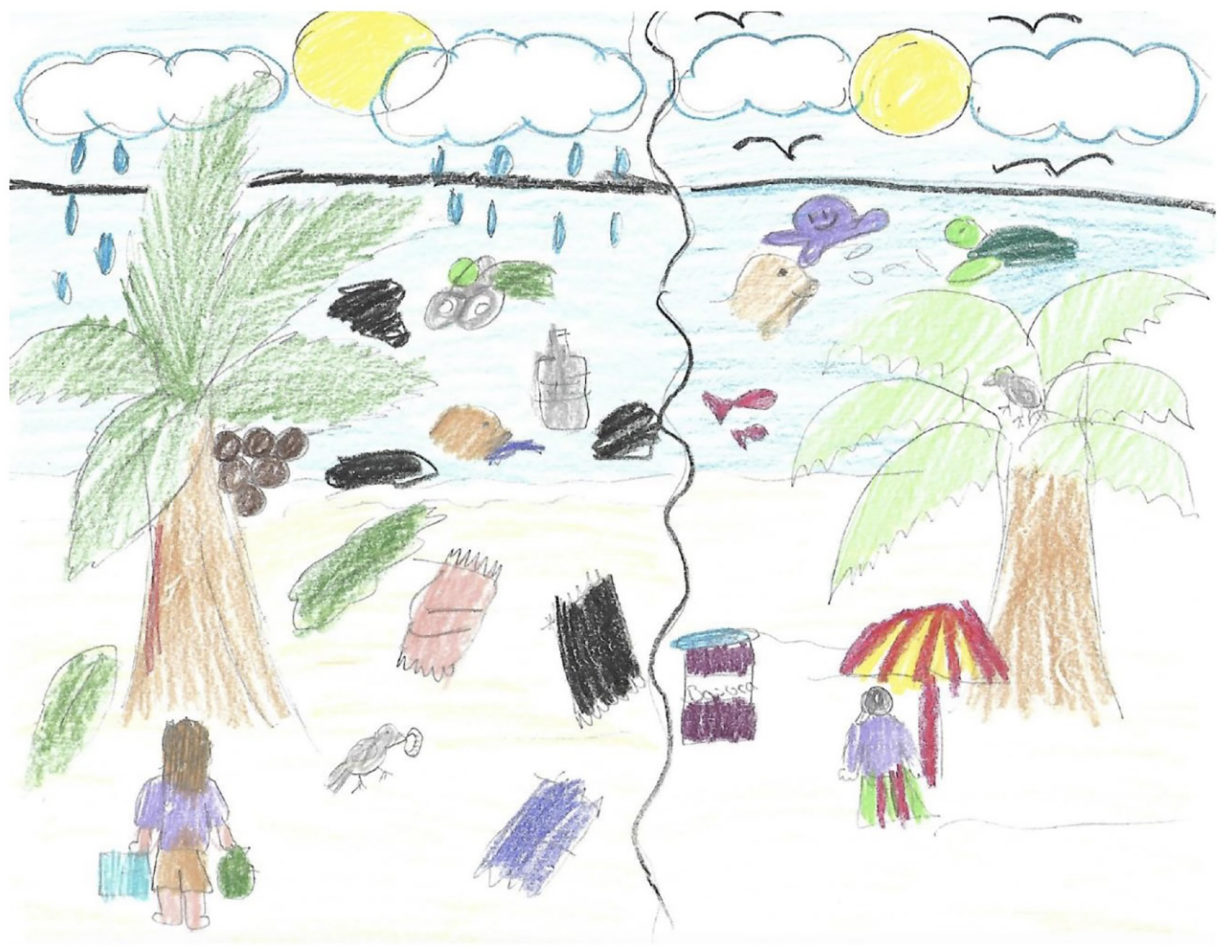
Drawing by participant #14, 14-year-old girl. Both a positive and a negative depiction of future.

*“I see two options in the future depending on how we act in the present. That is to say, if people decide to take care of the islands and protect them, we will have a situation like this* [the positive drawing]*, and it will continue to be preserved. In the other drawing I am showing what happens if the people mistreat it.”* (Participant #14, 14-year-old girl)

A subtheme of the Pollution category is the participants’ Understanding of Climate Change. This is because all participants who were able to describe their idea of climate change thought pollution was a central cause, most often littering. They frequently asserted that littering directly causes climate change, a perspective shared by 24 of the participants (80%), although an equal proportion of participants were also able to identify other types of pollution that contributed to climate change, such as emissions from factories and vehicles. They also used the word *contaminación* (pollution, contamination) freely and broadly, and linked it to all human harms to the environment.

*“As we know well. Today, climate change is a reality, there is no denying it, and it is caused by us. And as long as more people go taking garbage and throwing it in our oceans, there is going to be more climate change*…*A long time ago, [climate change] didn’t exist and it is because of the litter, because of the aerosols, because of the factory fuels, because of the factory smoke, because of the cars. Also, because of, also cow excrement is a pollutant.”* (Participant #14, 14-year-old girl)

When asked the message she would like to share with adults about climate change, the 8-year-old girl who drew Figure 9 replied,

*“I’d like to say*…*that please don’t litter on planet earth, because if not, it’s going to get worse. And well, if they keep doing it, well, well, it’s going to cause earthquakes, tsunamis, earthquakes, storms, tornados. And imagine that, if one day you cut down the trees every day, every day and the trees run out. We wouldn’t have fresh air and we would be breathing gases and things like that.”* (Participant #10, 8-year-old girl)

Twelve participants (40%) argued that, to combat climate change, the community should engage in campaigns to teach people not to litter, and highlighted parents’ responsibility to teach this to younger generations. The same proportion of participants (40%) also suggested walking, riding bikes, or using electric vehicles instead of gas-burning cars. These recommendations fell under the subtheme of Taking Action. The urgency for these recommendations was often layered with feelings of sadness and loss or frustration:

*“In the case of adults and those of us who are young people, we can encourage the little ones and those of the whole community to*… *um, learn how to use*… *recycling, caring for nature, not throwing garbage wherever because that is why there are trash bins*… *um*… *Yes, some people, if they ride a bicycle so as not to be using, so that gas does not come out of cars and those polluting things. I say that we must raise awareness, have more, if we don’t know, investigate more on the subject about the consequences, that we are going to have, in the*… *it could be now or in the new generations that*… *we should reconsider. Because if not, these islands are, they are wonderful. You can say a paradise. And if we don’t take care of it now, what will the new generations receive, if we don’t do things right, the new generations won’t do it either. And little by little the islands will be lost.”* (Participant #19, 16-year-old girl)

Some participants made comments that layered the theme of Pollution onto the theme of Nature, Community, and Identity. Four participants (13%) asserted that a conservation mentality is engrained in the people of San Cristóbal.

*“Everyone has the*…*or, almost everyone teaches us*… *according to me, the custom that we have to take care of our animal species in the Galápagos. No littering and all that stuff. Because we are always taught in school. that is, we are always taught that we have to be more conservationist*… *[In the future, I think the island will be] very modernized. But I hope that everyone applies that, I don’t know, that how shall we say, culture of*… *of conservationism that they have always taught us and do not occupy natural spaces.”* (Participant #1, 15-year-old boy)

#### Emotional responses

Many Emotional Responses that overlapped with other themes are discussed above. Individually, however, these responses also emerge as an independent theme with subthemes, including Sadness and Loss, Frustration and Anger, and Fear or Worry related to climate change. Sadness and Loss was the most common of the three subthemes, accounting for 44% of all Emotional Responses. The statements in this subtheme describe a very bleak future, but the participants always delivered this objectively sad information in a very calm and matter-of-fact manner. One 14-year-old girl drew a future of San Cristóbal (Figure 11) in which the island is totally abandoned. The water is littered with trash and microplastics, and she said humans abandoned the location because it “*doesn’t have flora, not a bit of fauna either, [so] there would be no tourism there*” (Participant #11, 14-year-old girl). When the interviewer said the drawing seemed sad, the participant responded, “*It’s realistic, [*…*] it’s what we are living. We are not living in a world of happiness”* (Participant #11, 14-year-old girl).

**FIGURE 11:**
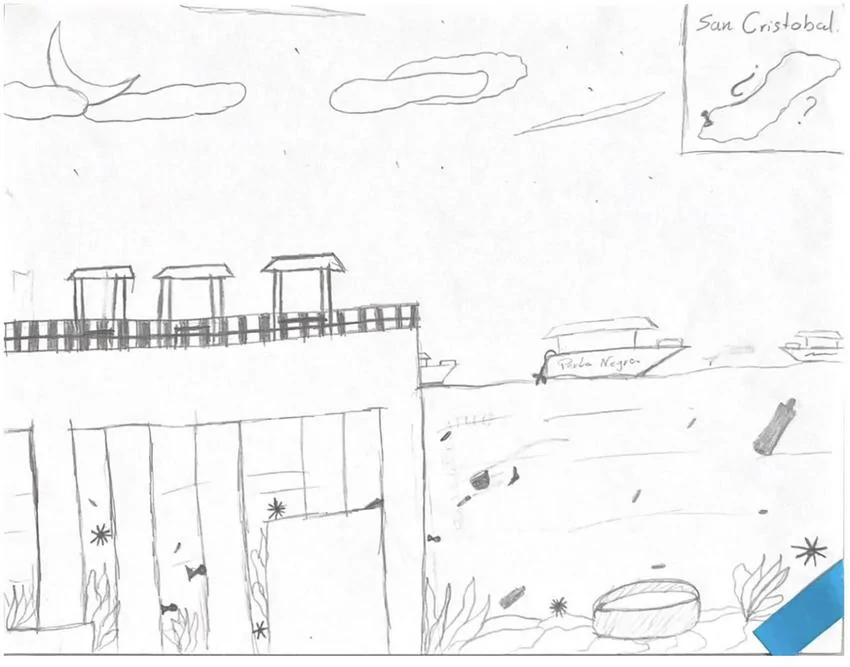
Drawing by participant #11, 14-year-old girl. Negative depiction of future.

Many other participants (25/30, 83%) expressed Sadness and Loss when considering the future and climate change. A 12-year-old girl drew a picture of the future in which sea level rise and littering killed many animals and prevented humans from being able to enter the water safely (Figure 12). She reflected,

**FIGURE 12:**
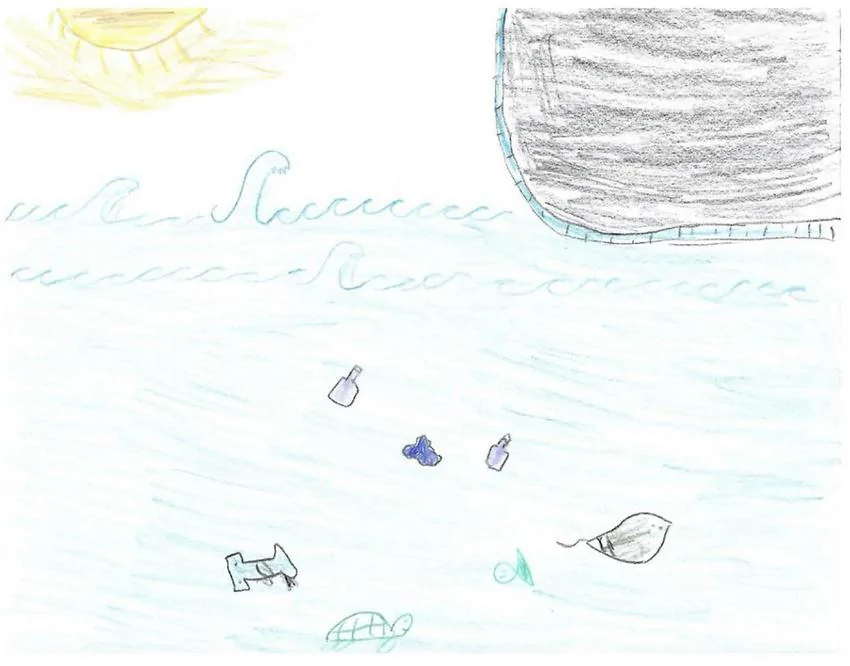
Drawing by participant #2, 12-year-old girl. Negative depiction of future.

*“Some things that I think about, when I go see the sunsets and I see that one day this is going to end*…*These islands are like the treasure here. And one day there will be none of this. And then our memories will stay in the past.”* (Participant #2, 12-year-old girl)

An 11-year-old girl, described her drawing of the future of the island (Figure 13):

**FIGURE 13:**
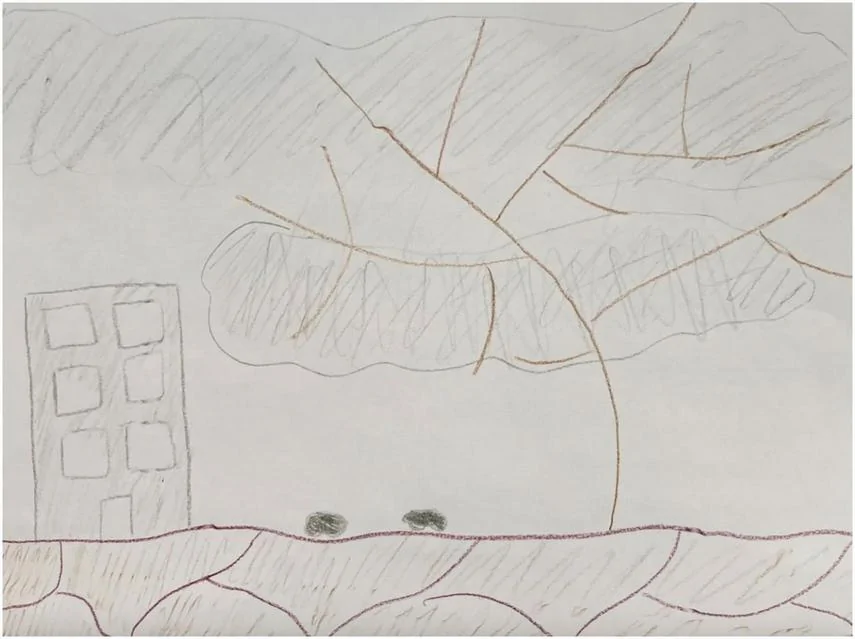
Drawing by participant #3, 11-year-old girl. Negative depiction of future.

*“The trees will no longer have more*… *more leaves and everything will be littered. The clouds will no longer be white; they will be gray and there won’t be much sun anymore. Because everything will be polluted and everything is dark. And the sky is all contaminated and. And the ground will be like this, cracked and dry. And there will be bad, bad drought, and there will be no more water.”* (Participant #3, 11-year-old girl)

Expressions of Fear and Worry were also very common and arose in 70% of the interviews (21 participants), especially when they discussed the impact that climate change will have on human beings, not only on animals.

*“[Climate change] makes me sad. And it also scares me because, I mean, it hurts people and animals more than anything. It will destroy everything, practically everything.”* (Participant #20, 15-year-old girl)

*“There are many people, well, many young people, and in my case, I would not like, for example, to have children at the moment. One, because I am young, and two, also because I think that. if there are people who are not going to educate their children, this is going to happen and they are going to suffer all these things. So I think it’s going to be something very awful for my children that they suffer things from climate change that are going to come very soon.”* (Participant #16, 15-year-old girl)

Thirteen participants (43%) also expressed Frustration and Anger with respect to climate change, especially toward generations of adults who they feel have not taken enough action.

“*A message for adults about climate change*…*Instead of just giving words, why don’t you give actions? In other words, they say, ‘We are going to do it,’ but they don’t, they just talk, they just talk all the time, they just want to talk and not listen to others. And as an adolescent, young person, child, you can’t say anything because they are the authority at that moment. So they don’t want or do not want to hear an opinion from someone younger than them and they want to be superior*.” (Participant #11, 14-year-old girl)

*“Well, my feelings are, like, a little angry and a little sad. Because despite. despite what they know is going to happen. That there will be a day when they cannot get into the water. One day there is going to be a big, big elevation of the sea. And it can cause harm to many, to different people here in the town that are near the water. And also, the animals that I like, I mean, they would die. And the feeling of anger is because they know what is going to happen and they do not do something to help.”* (Participant #2, 12 year-old girl)

On the other hand, there were some participants who did not feel particularly moved by climate change. For example, a 15-year-old boy explained, *“Climate change doesn’t cause any special feelings in me”* (Participant #24). He was the only participant who shared this neutral position on the topic, although there were two additional participants who felt unable to provide a general definition of climate change and therefore could not describe their direct feelings on the topic. Others endorsed feeling comforted by the culture of conservation on the island and felt that humans will successfully protect the environment from the worst impacts of climate change (6/30, 20%). This perspective led to drawings like Figure 14, which reveals a future with increased infrastructure along the city’s main waterfront, but the expanded buildings are still mingling harmoniously with nature. One participant even wrote a small blurb at the base of her drawing (Figure 15), emphasizing the positive changes she expects to see: *“I imagine a San Cristóbal with less garbage in the ocean and more populated and that is equally calm and safe, with more marine species”* (Participant #30, 15-year-old girl). These statements applied to the subtheme of Optimism. Although these sentiments could be interspersed within the same interview with other more negative Emotional Reponses, a total of 11 participants (37%) shared optimistic statements.

**FIGURE 14:**
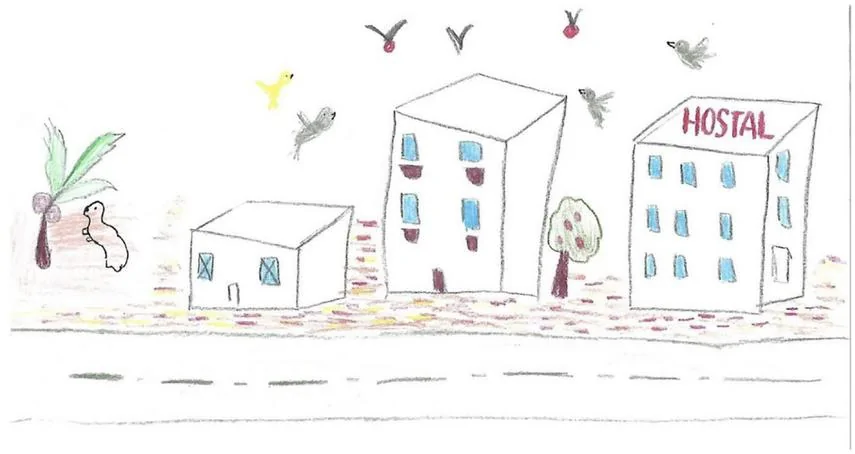
Drawing by participant #1, 15-year-old boy. Positive depiction of future.

**FIGURE 15:**
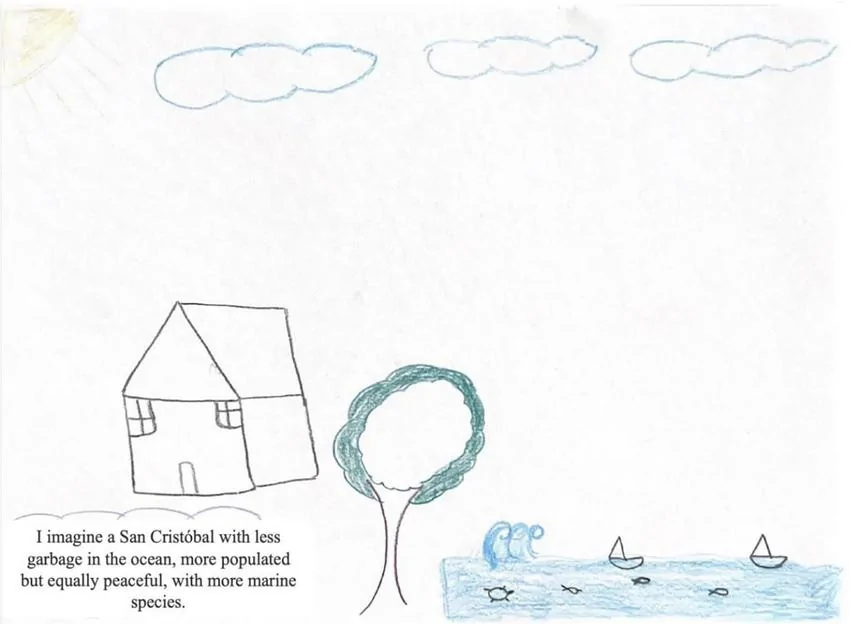
Drawing by participant #30, 15-year-old girl. Positive depiction of future (Her handwritten text at the bottom of the drawing is replaced with a typed translation in English).

Participants varied in how frequently they endorsed reflecting on climate change and their concerns about its impacts. Nine participants (30%) expressed that they either think about it very frequently or are reminded of it often.

*“[I think about climate change] constantly. Because it is something that is happening a lot and you see so much of how it’s being demonstrated in earthquakes, that some countries have been flooded, that the sea level is rising. That animals are dying, so it’s something that you see very constantly.”* (Participant #1, 15-year-old boy)

*“[I think about climate change] almost always. Because on the Internet we can see, for example, anything in videos that YouTube recommends. It shows you about what is currently happening.”* (Participant #6, 12-year-old boy)

Three participants (10%) said it was quite rare that they thought about climate change; one of them explained, “*I don’t have much time to think about so many things that go through my mind*” (Participant #11, 14-year-old girl). Most other participants (16/30, 53%) said they tended to think about climate change when they see certain problems in their environment, are part of discussions about climate change, or when the topic arises at school or in the news.

*“Not every day, but when I see someone do something bad. Sometimes I even do some bad thing, but I remember that I shouldn’t do it, because of the teachings they have given me and the care I have to take in the Scouts; we value nature very much, and the life of each being.”* (Participant #19, 16-year-old girl)

*“[I think about climate change] when it’s a subject I study at school.”* (Participant #24, 15-year-old boy)

Overall, there was variation in the amount of emotional expression across genders.

Expressions of emotion were coded in girls’ interviews 1.9 times more often than in boys’ interviews. Sentiments of Sadness and Loss appeared 2.4 times as often in the girls’ interviews compared to the boys. Frustration and Anger appeared about three times as often amongst the girls. Fear or Worry occurrences were about equal between the genders, and the frequency of these thoughts was equal as well. On average, the Scouts shared expressions of Sadness and Loss about twice as often as the other participants, although the divers expressed Frustration and Anger more than other groups (1.3 time more than Scouts, and 2.8 times more than those recruited through the church). The two participants who were recruited from the Evangelical church community expressed Fear and Worry more often in their interviews than the other participants, with statements arising in their interviews about twice as often on average.

## Discussion

This project aimed to understand children’s perspectives, emotions, and understanding of climate change in San Cristóbal, Galápagos, an island community that will be highly vulnerable to climate change impacts. There have been limited studies on youth perspectives on climate change in island communities in LMICs, although the few investigations that have been conducted in locations such as Fiji and the Philippines found that climate change was a common and compelling concern for adolescents (Berse, 2017; Sciberras and Fernando, 2022; Reyes et al., 2023). Similar to the children in this study, adolescents in Fiji recommended community education campaigns and expressed frustration with adults (Scott-Parker and Kumar, 2018). In a survey of Generation Z Filipinos aged 18–26, they found that climate change anxiety was associated with a significant decrease in overall mental health, as measured by the MHI-38 global scale of Psychological Distress (Reyes et al., 2023). The relative lack of literature on climate anxiety among children in LMICs was one of the motivations for this study, especially given the potential implications on children’s mental health outcomes. This concern is reinforced by a longitudinal study in Australia that found that adolescents with persistently high climate change-related worry as they aged from age 10 to 18 had more depression symptoms than those with lesser worry (Sciberras and Fernando, 2022). It was therefore important to determine if children were experiencing emotional repercussions of climate change that may warrant intervention in San Cristóbal, especially considering the future projected impacts of climate change in Galápagos.

The findings from this study revealed prominent themes in the participant’s responses: Animal Concerns, Nature, Community, and Identity, and Pollution, with Emotional Responses overlapping with all prior themes. Amongst the responses citing Animal Concerns, every participant identified an animal on the island that was important to them and was able to explain why, 97% expressed concern that animals would be harmed or experience reductions in numbers in the future, and 77% shared emotional responses when discussing these concerns for animals. It was important to note that the participants were directly asked if there was an animal that is important to them, which may have primed them to consider animals in the drawing process or in later discussion. Nevertheless, the strength and poignancy of the quotes suggests that their statements about animals reflect genuinely-held beliefs.

Under the second theme of Nature, Community, and Identity, 90% of participants expressed a sense of Peace and Belonging in Nature and 83% expressed Sadness and Loss when considering damages to the natural environment due to climate change. The strength of the participants’ connections to animals and to the natural environment may explain why they spoke with this sense of sadness when considering future losses. This may be relevant when considering that the Scouts expressed more sentiments coded to the Nature, Community, and Identity category than other participants, and they subsequently were found to have greater expressions of Sadness and Loss as well. The Scouts also had a higher proportion of negative drawings (14/21, 67%) compared to the divers (3/7, 43%). The Scouts may have had a stronger fostered connection with the natural world due to their weekly meetings: led by naturalist guides, these Scouts participate in activities in nature such as camping and swimming, and are taught to respect and care for the environment and to advocate for others to do the same. The divers also experienced activities in nature, namely through scuba diving trips, although their excursions were far less-regularly scheduled and far less numerous than Scout gatherings. While past studies have shown that positive experiences in nature are associated with pro-environmental attitudes (Arnold et al., 2009; Buttigieg and Pace, 2013; Corner et al., 2015), our data suggest other factors could contribute to shaping perceptions: the Scouts also included more references to Family Interactions in Nature, suggesting that an appreciation of the natural world may be a value promoted in their households.

Under the Pollution theme, 97% of participants raised the topic of littering in their interviews and it was featured in 40% of the drawings. Notably, 80% of participants considered litter to be a direct causal factor of climate change, the same proportion of participants who were able to correctly emphasize the role of factory and vehicle emissions. In the academic year prior to the interviews, all schools on the island were required to teach about climate change at all grade levels as part of a new Contextualized Curriculum for Galápagos (Ministerio de Educación, 2021). The teaching was not standardized, however, so children had varying experiences and levels of exposure to the topic.

This may explain why nearly all the children in our sample (28/30, 93%) were able to offer a general description of climate change, although it was often not a perfect scientific explanation. Participants’ perseveration on litter is understandable given the visible nature of its daily presence on the island, but also due to pervasive messaging they have received on this topic; campaigns to discourage littering are common in the community, and the children shared that they received this messaging in formal (e.g., school) and informal settings (e.g., during Scout activities or clean-up dives, where scuba divers collect marine trash in the bay).

Overall, 83% of participants expressed Sadness and Loss, 43% expressed Frustration or Anger, and 70% expressed Fear or Worry. These rates of concern are drawn from a younger sample than most investigations on this topic, but seem to align with recent survey data that found 84% of youth aged 16–25 were at least moderately worried about climate change, and 59% were very or extremely worried (Hickman et al., 2021). Similarly, in a study conducted among fifty 10—12-year-olds in Colorado, 82% of participants expressed sadness, fear, or anger with respect to environmental issues (Strife, 2012). However, our findings from Galápagos contrasted with some data from an older Swedish survey conducted in 2009 (Ojala, 2012). In a sample of 350 children and young adults, 70% of children and 38% of adolescents said that they worried ‘little or not at all’ about climate change. In open-ended components, the author found that many participants deemphasized the issue of climate change, which did not occur in any of the interviews in San Cristóbal. They also found that adolescents and young adults rarely engaged in “hyperactivation,” which included feelings of fatalism, helplessness, or rumination on worry (Ojala, 2012). In contrast, our participants in San Cristóbal frequently shared statements of fatalism and frustration related to helplessness. Various factors may affect these differences; our study notably took place 13 years later than the Swedish study, in a location renowned for its natural environment—one whose economy is highly dependent on offering tours to a National Park that comprises 97% of the available land.

Thirty percent of participants in this study endorsed thinking about climate change “always,” “constantly,” or “all the time.” More than half (53%) reported that they think about climate change when they see someone engaging in environmentally harmful behavior, or when the topic arises in school, at home, or in the news. Three participants (10%) reported that they rarely think about climate change at all. This aligns with data from Australia, where a survey of more than 1500 students aged 14 to 23 found that 37% of participants feel anxious about climate change on a weekly basis (ReachOut, 2019).

The frequency of Frustration and Anger expressed among participants aligns with recent survey data related to youth’s dissatisfaction with global responses to climate change. In an international study of more than 10,000 youth in 2021, negative thoughts and worry about climate change were correlated with feelings of betrayal and negative beliefs about governmental responses to climate change (Hickman et al., 2021). For instance, a majority of these participants agreed with negative statements about government performance. In the Australian survey mentioned above, 77% of respondents said that they did not think young people’s concerns about climate change were being suitably addressed (ReachOut, 2019). In focus groups conducted with adolescents aged 14–18 on the island of Fiji, participants expressed frustration at feeling dismissed by adult family members, and said that due to cultural norms they could not feel comfortable asserting the issue of climate change with their elders (Scott-Parker and Kumar, 2018).

The girls in our study shared Emotional Expressions more often than the boys despite both genders having roughly equal endorsements of Personal Connection to Animals and Peace and Belonging in Nature. It is possible that the girls in our sample genuinely have stronger feelings with respect to climate change than the boys do: 83% of the girls drew negative depictions of the future compared to 38% of the boys, which may support the notion that the girls are more troubled by climate change and environmental harms than the boys in our sample. It is also possible that the girls in our sample were more adept at expressing their emotions on the topic than the boys, and thus providing more responses characterized as Emotional.

Overall, 63% of the drawings shared negative perspectives on the future while 17% were neutral and 17% were positive. Eleven participants (37%) shared optimistic statements about the future, although they were often intermixed with more negative Emotional Responses. This suggests that the children had predominantly negative views regarding the future of the environment, although a substantial minority did share hopeful sentiments. This may align with data from the Australian survey referenced above, in which 80% of participants agreed with the statement, “climate change is going to diminish my quality of life in the future” (ReachOut, 2019). In a study conducted among fifty 10–12-year-olds in Colorado, when children were asked to draw what they thought the planet would look like in 100 years, most (72%) shared a pessimistic outlook for the future of the planet, and many drew “apocalyptic” depictions (Strife, 2012). Additionally, in a recent international survey on climate anxiety published in 2021, 75% of respondents endorsed that they think the future is frightening (Hickman et al., 2021). Some may argue that the children in this study are merely expressing realistic perspectives given the current threats of climate change. Past studies have also shown that children are susceptible to the “emotional contagion” of doom and gloom media messaging related to the environment (Kelsey, 2016).

The participants who had parents working in scientific or naturalist fields tended to express more negative ideas of the future, with 75% (9/12) drawing negative depictions. This may be because parents in scientific fields or who work in the National Park are more acutely aware of environmental harms due to their profession, and may share information about these harms with their children. In contrast, the participants whose parents had jobs most likely to be impacted directly by climate change (Farmers, Captains/Fishermen, Tourism Agency), had more neutral and positive drawings: of the 11 drawings, 37% were negative, 36% were neutral, and 18% were positive. Although these jobs are likely to be affected by climate change, people in these professions may have less access to scientific information and therefore may be less aware of future threats. Prior studies have established that parents’ environmental values and perspectives are influential in their children’s awareness of climate change and engagement in pro-environmental behavior (Stevenson et al., 2019; Crandon et al., 2022; Gong et al., 2022).

### Limitations and future directions

The main methodological limitation for this study was an inability to assess the SES of communities or participants beyond parental profession, which acted as a descriptor. Despite the inclusion of scuba divers from a separate GERA initiative that intentionally recruited low-SES student participants, we could not compare across participants using SES.

The sample in this study has limited generalizability, both within the island of San Cristóbal, and with respect to global populations of children. Most of the participants (21/30, 70%) were part of a Scout troop, a group led by two naturalist guides that intentionally fosters an attitude for environmental protection. Seven additional participants were part of a scuba diving initiative where they were taught to appreciate and honor the underwater world. The remaining two participants were related to a naturalist guide. Therefore, all the participants had connections to the environment that may be outstated compared to the average child on the island. The team intended to recruit children who live in agricultural regions of the highlands and children who are part of the indigenous Salasaca community, in order to appreciate their unique perspectives, but recruitment attempts through GERA’s community partners were not successful. The age distribution of this sample was also unevenly distributed across the range of 8–16, with 43% of the participant pool either age 15 or 16. Younger children were not as well represented. Additionally, the environmental perspective of children in in Galápagos—a unique archipelago that is world-famous for its species and marine ecosystems—likely cannot be generalized to represent children living in other locations.

This study did not use any formal measures of climate anxiety because such tools have not yet been created or validated for children. Recommendations for future directions include using this qualitative data to help inform the creation of a formal instrument to measure climate anxiety and related concepts such as eco-grief in children. This data was also collected with the intention of informing educational initiatives and intervention plans to better support the emotional wellbeing of children in the Scouts and diving group. The limited generalizability will not interfere with this aim, as the interventions will be tailored to the participants in this study. Another recommendation is to determine the most effective interventions for children experiencing climate anxiety, as well as climate change adaptation measures that island governments can take to best support children.

## Conclusion

This study revealed that amongst a limited sample in San Cristóbal, Galápagos, children are experiencing a strong connection to the natural world. Young people in this community report identifying with local animals and feeling a sense of peace and belonging in nature. Most of the participants expressed sadness and loss when considering the potential impact of climate change on this natural environment, along with common expressions of fear, worry, and frustration. The participants were particularly preoccupied with litter and pollution, and the majority envisioned a negative future for the island in their drawings. GERA will be using these findings to engage in community-oriented work to better support the children and adolescents on the island, including teaching emotional coping techniques, offering interactive educational initiatives, and facilitating opportunities for youth activism.

## Data Availability

The original contributions presented in the study are included in the article/Supplementary material, further inquiries can be directed to the corresponding author.
