# Supplementary material for: Investigating perspectives and emotions on climate change among children and adolescents in San Cristóbal, Galápagos
Source: Front Psychol. 2026 Oct 6;17:1935891. doi: 10.3389/fpsyg.2026.1935891 (PMC13680255; doi:10.3389/fpsyg.2026.1935891)
Supplement: Supplementary file 1 [file Supplementary_file_1.docx]

**Supplementary Materials**

Submission: Investigating Perspectives and Emotions on Climate Change Among Children and Adolescents in San Cristóbal, Galápagos

Authors: Moffitt, Kennedy, Malefakis, Pinto-Martin

See below for Appendices

# Appendix A: Interview Guide (Original, Spanish)

*La entrevista a continuación comienza con algunas preguntas demográficas iniciales, es seguida por una actividad de dibujar y finaliza con una serie de preguntas de la entrevista.*

La entrevistadora se presentará y hablará un poco sobre de dónde es. Entonces ella preguntará: "¿Puedes decirme dónde vives?"

- “¿Puedes decirme algo importante sobre el lugar en que vives?”
- “¿Puedes dibujar donde vives?”
- “¿Cómo es un día normal para ti?”
- “¿Que te gusta hacer para divertirte?”
- “¿Cuándo pasas tiempo al aire libre? ¿Cuán a menudo haces eso?”
- “¿Qué te gusta hacer afuera?”
  - “¿Puedes contarme una historia sobre una experiencia (memorable) que hayas tenido al aire libre? ¿Puedes contarme una historia sobre un momento en que te divertiste afuera?”
  - “¿Qué haces cuando quieres estar afuera?”
- “Cuando sales y estás afuera, ¿qué escuchas, ves, hueles (y saboreas)?”
- “¿Cómo te sientes acerca de pasar tiempo al aire libre? ¿Qué piensas acerca de pasar tiempo en la naturaleza?”

La entrevistadora hará la transición a **la actividad de dibujar**, pidiéndole que dibuje “cómo cree que se verá San Cristóbal dentro de 100 años.” Esto lo orientará a pensar en el futuro.

- Pregunte, “¿En qué estabas pensando cuando hiciste este dibujo?”
- (Deje en claro que el dibujo no tiene que ser hábil o artístico!)

A continuación, la entrevista pasa a preguntas sobre **aspectos concretos del entorno del niño y/o adolescente: Los animales**

- Pregunte: “¿Hay algún tipo de animal que viva en San Cristóbal que sea importante para tí?”
  - Si dice que no, pregúntale si hay algún animal que vea con frecuencia.
  - “¿Dónde vive ese animal?”
  - “¿Cómo está cambiando el hábitat de ese animal?”
  - Como pregunta de seguimiento: “¿Estás preocupado por este animal?”

A continuación, el entrevistador pasa a **hitos concretos** en el futuro del niño:

- “¿Te gustaría vivir en la isla después de graduarte de la escuela secundaria?”
- “¿Cómo crees que será la isla cuando tengas 20 años? ¿y cuando tengas 30 años?”
- “¿Te gustaría tener hijos o formar una familia? En caso afirmativo, ¿crees que te gustaría formar una familia aquí en la isla?”

A continuación, la entrevistadora puede hacer una pregunta directa sobre **el cambio climático** si aún no ha surgido de forma natural:

- “¿Has oído hablar del cambio climático?”
  - En caso afirmativo:
    - "¿Cómo describirías el cambio climático a alguien que nunca ha oído hablar de el?" o “¿Cómo lo definirías?”
    - “¿Qué crees que podría estar causando el cambio climático?”
    - “¿Dónde/cómo se enteró de eso?”
      - Si aprendieron sobre el cambio climático en la escuela o mediante actividades extracurriculares: “¿Qué aprendiste?”
    - “¿Puedes decirme tus sentimientos sobre el cambio climático? ¿Qué piensas con respecto al cambio climático?"
    - “¿Con qué frecuencia te encuentras pensando en el cambio climático?”
    - “¿Tienes algún mensaje para los adultos acerca del cambio climático?”
    - “¿Es algo sobre lo que deberíamos estar haciendo más? ¿Qué deberíamos estar haciendo?”
    - “¿Crees que es algo de lo que deberíamos estar preocupados o no? ¿Por qué?"
    - “Si los adultos estuvieran haciendo lo correcto con respecto al cambio climático, ¿cómo sería eso? ¿Te sentirías diferente?”
    - “Si se garantizara la seguridad de todos los animales, ¿cómo se sentiría entonces sobre el cambio climático?”
  - Si no está familiarizado con la idea del cambio climático, intente preguntarle sobre este concepto de otras maneras:
    - “¿Has oído hablar del calentamiento global?”
    - “¿Has oído que las temperaturas, las tormentas y la precipitación en todo el mundo están cambiando? ¿Y aquí en San Cristóbal?”
      - “¿Puedes explicar lo que has oído?”
    - “¿Has oído cómo las acciones de los humanos durante muchos años han cambiado el medioambiente? ¿Has oído hablar de cómo las ciudades, los pueblos, las fábricas y las máquinas están afectando el medioambiente?”
      - “¿Puedes explicar lo que has oído?”
    - “¿Están los humanos cambiando la salud del planeta? ¿Cómo?”

Pregunte las últimas preguntas:

- “¿Tienes otra preocupación sobre el medio ambiente? ¿Hay otro sentimiento sobre el mundo natural que te gustaría compartir? ¿Hay algún cambio en la naturaleza o en los animales de la isla que te esté preocupando? Por favor explique."
- "¿Hay algo más que quieras compartir que no hayamos hablado?"
- "¿Qué piensas sobre la conversación que acabamos de tener?"

# Appendix B: Interview Guide (Translated, English)

*The interview below begins with some initial demographic questions, is followed by a drawing activity, and ends with a series of interview questions.*

Interviewer begins by introducing themselves and talking a bit about where they are from. Then they will ask: “Can you tell me about where you live?”

- “Can you tell me something important about where you live?”
- “Can you draw where you live?”
- “What is a normal day like for you?”
- “What do you like to do for fun?”
- “When do you spend time outside? How often do you spend time outside?”
- “What do you like to do outside?”
  - “Can you tell me a story about a (memorable) experience you had outside/outdoors? Can you tell me a story about a time you had fun outside?”
  - “What do you do when you want to be outside?”
- “When you go outside, what do you hear, see, smell (and taste)?”
- “How do you feel about spending time outside? How often do you do that? What do you think about spending time in nature?”

Then move to the **draw-and-explain activity**, asking them to “draw what they think San Cristóbal will look like in 100 years.” This will get them oriented to thinking about the future.

- Ask, “What were you thinking when you drew this picture?”
- (Make it clear that the drawing doesn’t have to be skillful or artistic!)

Next, the interview moves on to questions about **concrete examples in their environment: Animals**

- “Is there a type of animal that lives in San Cristóbal that is important to you?”
  - If they say no, ask if there is an animal that they see frequently.
  - “Where does that animal live?”
  - “Do you think that animal’s habitat is changing? If so, how?”
  - As a follow-up question: “Are you worried about this animal?”

Next, move to **concrete milestones in their own future:**

- “Do you want to live on the island after you graduate high school? Why/why not?”
- “What do you think the island will be like when you are 20 years old? What about when you are 30 years old?”
- “Would you like to have children or start a family?”
  - If yes: “Do you think you want to start a family here on the island?”

Next, can ask **a direct question about climate change** if it hasn’t already come up naturally:

- “Have you heard of climate change? “
  - If yes:
    - “How would you describe climate change to someone who has never heard of it?” or “How would you define it?”
    - “What do you think might be causing climate change?”
    - “Where did you hear about it?”
      - If they learned about it in school or through extracurricular activities: “What did you learn?”
    - “Can you tell me your feelings about climate change? What do you think about it?”
    - “How often do you find yourself thinking about climate change?”
    - “Do you have a message for adults about climate change?”
    - “Is it something we should be doing more about? What do you think we should do?”
    - “Is it something we should be worried about? Why?”
    - “If the adults were doing the correct thing with respect to climate change, what would that look like? How would you feel differently?”
    - “If the safety of all animals were guaranteed, how would you feel about climate change then?”
  - If they are not familiar with climate change, try asking about this concept in a less explicit way:
    - “Have you heard of global warming?”
    - “Have you heard that temperatures, storms, and rainfall all over the world are changing? What about here in San Cristóbal?”
      - “Can you explain what you have heard?”
    - “Have you heard how humans’ actions over many years have changed the environment? Have you heard about how human cities, towns, factories, and machines are affecting the environment?”
      - “Can you explain what you have heard?”
    - “Are humans making the planet less healthy? How?”

Ask the final questions:

- “Are there any other feelings or concerns about the natural world that you’d like to share? Are there any changes to nature or animals on the island that are worrying you? Please explain.”
- “Is there anything else you want to share that we didn’t talk about?”
- “What do you think about the conversation we just had?”
